# Sortilin Fragments Deposit at Senile Plaques in Human Cerebrum

**DOI:** 10.3389/fnana.2017.00045

**Published:** 2017-06-07

**Authors:** Xia Hu, Zhao-Lan Hu, Zheng Li, Chun-Sheng Ruan, Wen-Ying Qiu, Aihua Pan, Chang-Qi Li, Yan Cai, Lu Shen, Yaping Chu, Bei-Sha Tang, Huaibin Cai, Xin-Fu Zhou, Chao Ma, Xiao-Xin Yan

**Affiliations:** ^1^Department of Anatomy and Neurobiology, Central South University School of Basic Medical ScienceChangsha, China; ^2^Cancer Research Institute, Central South UniversityChangsha, China; ^3^School of Pharmacy and Medical Sciences, Sansom Institute, Division of Health Sciences, University of South AustraliaAdelaide, SA, Australia; ^4^Department of Human Anatomy, Histology and Embryology, Institute of Basic Medical Sciences, Neuroscience Center, Chinese Academy of Medical Sciences, School of Basic Medicine, Peking Union Medical CollegeBeijing, China; ^5^Department of Neurology, Xiangya Hospital, Central South UniversityChangsha, China; ^6^Key Laboratory of Hunan Province in Neurodegenerative Disorders, Xiangya Hospital, Central South UniversityChangsha, China; ^7^Department of Neurological Sciences, Rush University Medical CenterChicago, IL, United States; ^8^Laboratory of Neurogenetics, National Institute on Aging, National Institutes of HealthBethesda, MD, United States

**Keywords:** Alzheimer’s disease, amyloid deposition, neuritic plaques, synaptic pathology, Vps10p family proteins

## Abstract

Genetic variations in the vacuolar protein sorting 10 protein (Vps10p) family have been linked to Alzheimer’s disease (AD). Here we demonstrate deposition of fragments from the Vps10p member sortilin at senile plaques (SPs) in aged and AD human cerebrum. Sortilin changes were characterized in postmortem brains with antibodies against the extracellular and intracellular C-terminal domains. The two antibodies exhibited identical labeling in normal human cerebrum, occurring in the somata and dendrites of cortical and hippocampal neurons. The C-terminal antibody also marked extracellular lesions in some aged and all AD cases, appearing as isolated fibrils, mini-plaques, dense-packing or circular mature-looking plaques. Sortilin and β-amyloid (Aβ) deposition were correlated overtly in a region/lamina- and case-dependent manner as analyzed in the temporal lobe structures, with co-localized immunofluorescence seen at individual SPs. However, sortilin deposition rarely occurred around the pia, at vascular wall or in areas with typical diffuse Aβ deposition, with the labeling not enhanced by section pretreatment with heating or formic acid. Levels of a major sortilin fragment ~15 kDa, predicted to derive from the C-terminal region, were dramatically elevated in AD relative to control cortical lysates. Thus, sortilin fragments are a prominent constituent of the extracellularly deposited protein products at SPs in human cerebrum.

## Introduction

Senile plaques (SPs) were first described by Blocq and Marinesco in examination of silver stained brain samples from elderly epileptic patients (Critchley, 1929). The lesion was observed subsequently in the brains of elderly with and without dementia by other pioneer neuroscientists (e.g., Redlich, Alzheimer, Persini, Oppenheim, Fisher, Cajal), and it was Simvhowisz who named the pathology as “senile plaques” (Critchley, 1929; García-Marín et al., 2007; Ohry and Buda, 2015). Based on the silver preparation, it was also known by that time that SPs contained swollen neurites and some amorphous material—the former was named as dystrophic neurites (DNs) while the entire lesion as neuritic plaques. The term “amyloid plaques” was coined later by Divry who found Congo red stain of the amorphous material (Oĭfa, 1973). Electron microscopic studies and evidence from immunolabeling of presynaptic and neurotransmitter markers (e.g., synaptophysin (SYN), choline acetyltransferase, tyrosine hydroxylase, glutamate decarboxylase and vesicular glutamate transporters) in plaque-associated DNs suggest that they appear to be largely abnormal axons including presynaptic terminals (Luse and Smith, 1964; Gonatas et al., 1967; Struble et al., 1982, 1987; Walker et al., 1985; Masliah et al., 1991; Ferrer et al., 1998; Cai et al., 2010; Yan et al., 2014; Sadleir et al., 2016).

By the mid-1980s, β-amyloid peptides (Aβ) were identified from amyloid vasculature and parenchymal plaques in the human brain, marking a milestone in the history of research on cerebral β-amyloidosis relative to Alzheimer’s disease (AD; Glenner and Wong, 1984; Masters et al., 1985). Within a few years antibodies to Aβ became routine tools to stain SPs for definitive diagnosis of AD. Site-specific deposition of Aβ at cerebral vasculature, meninge and diffuse plaques in the gray and white matter (WM) was also confirmed (Allsop et al., 1986; Jellinger and Bancher, 1988; Yamaguchi et al., 1988; Braak and Braak, 1991; Braak et al., 2006). The discovery of Aβ as the key components of cerebral β-amyloidosis has since led to many other breakthroughs in the AD research field and beyond, as partially listed: (1) characterization of the amyloidogenic proteins, i.e., β-amyloid precursor protein (APP), β-secretase-1 (BACE1) and γ-secretase complex (Robakis et al., 1987; Wolfe and Haass, 2001; Vassar et al., 2009); (2) establishment of the genetic link of APP and presenilin mutations to familial AD (Shea et al., 2016); (3) engineering of transgenic animal models of AD (Hsiao et al., 1996; Borchelt et al., 1997; Oddo et al., 2003; Oakley et al., 2006); (4) development of cerebrospinal fluid (CSF) biomarkers and Aβ imaging techniques for antemortem diagnosis of AD (Andreasen et al., 2001; Mathis et al., 2002; Herholz and Ebmeier, 2011); and (5) conceptualization of the anti-Aβ therapy that has advanced from bench to bedside testing (Aisen, 2005; Yan et al., 2014; Karran and De Strooper, 2016). Notably, some blood proteins, heavy metals and lipoproteins accumulate around amyloid plaques (Coria et al., 1988; Rogers et al., 1992; Schwarzman et al., 1994; Eriksson et al., 1995; Kida et al., 1995; Watson et al., 1997; Burns et al., 2003; Wu et al., 2004; Garai et al., 2014; Cristóvão et al., 2016). Exploring additional plaque constituents, if any, may also help advance the understanding of plaque pathogenesis in the brain.

Variations in the vacuolar protein sorting 10 protein (Vps10p) family genes are recently shown to affect the risk of developing AD (Westergaard et al., 2004; Hermey, 2009). Variants in several loci of the sortilin-related receptor L1 gene (*SORL1*, also known as *SORLA, SORLA1 or LR11*) can increase the risk of AD (Rogaeva et al., 2007; Pottier et al., 2012; Wen et al., 2013; Felsky et al., 2014; Louwersheimer et al., 2015; Verheijen et al., 2016). Variants of single nucleotide polymorphisms (SNPs) of the sortilin related Vps10p domain containing receptor 1 (*SORCS1*) may also relate to AD (Reitz et al., 2011). The SNP rs17646665 of the sortilin gene (*SORT1*) is linked to a reduced risk of AD (Andersson et al., 2016). The Vps10p proteins belong to type I transmembrane proteins, which might subject to proteolytic processing (Avci and Lemberg, 2015). The gene-association data obtained from human populations raise an intriguing issue as to whether the expression of some proteins from this family, including their potential fragment products, might be altered relative to AD-type neuropathology.

With the setup of human brain banks, a preliminary batch of postmortem brains from Chinese donors became available for aging and AD-related studies (Zhu et al., 2015; Griffith et al., 2016). Using this resource, we conducted a pilot survey on the expression of the Vps10p members in the brain with commercial antibodies, and noticed that a sortilin C-terminal antibody visualized extracellular lesions appearing as amyloid plaques. Experiments were then carried out to verify this sortilin pathology, to profile its relationship with Aβ deposition and to identify the candidate peptide component seen deposited in the plaques.

## Materials and Methods

### Human and Rodent Brain Samples

Postmortem human brains were banked through the willed body donation programs, which existed with government (municipal police department and office of the Red Cross Society of China) and university approval to provide cadavers for teaching anatomy to medical students. Efforts are being taken to develop this platform for human brain banking as a part of the initiatives supporting China Brain Project (Yan et al., 2015; Poo et al., 2016). A subgroup of the elderly cases was recorded being dementia at the time of hospitalization for the care of terminal illnesses (Supplementary Table S1). Brain samples were assessed for AD type-neuropathology using sections from temporal, parietal, frontal and occipital lobes, with the extent of pathology (if present) scored according to Braak’s staging and the NIH guideline (Jellinger and Bancher, 1988; Braak et al., 2006; Montine et al., 2012). Correlated anatomical and biochemical studies were carried out using samples from three groups designated according to age and AD-type neuropathology: (1) Mid-age cases (*n* = 9) died of non-neurological diseases and free of Aβ/tau pathology in the cerebrum; (2) Aged cases (*n* = 9) with a history of dementia but Braak’s score of neurofibrillary tangle ≥ IV and the ABC amyloid score ≥ B, defined as AD group, and (3) Aged control group (*n* = 9) with no Aβ/tau pathology observed in the brain. It should be noted that, for comparative pathological analyses, additional aged cases with cerebral amyloidosis were included (Supplementary Table S1). Through this latter approach, brain samples/cases exhibiting a spectrum of AD-type neuropathology were used to allow a correlated morphometric analysis on Aβ and sortilin pathologies. Brains from sortilin knockout (−/−, *n* = 4), wildtype (+/+, *n* = 4) and C57BL/6 mice were used for the purpose of antibody validation (Ruan et al., 2016), with hemi-brains obtained after vascular rinse with cold saline then prepared for immunohistochemistry and immunoblot, respectively. The use of postmodern human brains and laboratory animals was approved by the Ethics Committees of Central South University Xiangya School of Medicine and Chinese Academy of Medical Sciences, Institute of Basic Medical Sciences (human brain study), and the Animal Ethic Committee of SA Pathology at Adelaide, Australia (mouse brain study), in compliance with the Code of Ethics of the World Medical Association (Declaration of Helsinki) and the National Institutes of Health Guide for the Care and Use of Laboratory Animals.

### Tissue Processing

Postmortem human brains were bisected and cut into ~2 cm-thick frontal slices. Slices from one hemisphere (opposite to handedness) were fixed in formalin for at least 1 week, with the slices from the other hemi-brain stored at −70°C. Temporal, frontal, parietal and occipital lobe blocks were then sampled from the fixed hemisphere, and either embedded with wax or placed in 30% sucrose in 0.01 M phosphate buffer, which were further prepared into paraffin (5 μm thick) or cryostat (40 μm thick) sections. The cryostat sections were collected in phosphate-buffered saline (PBS, 0.01 M, pH 7.2), with consecutive sections placed orderly into 24 wells each containing 4–6 sections/block with equal distance (24 × 40 ≈ 1000 μm). The sections were rinsed with PBS twice to remove the embedding medium, and stored in a cryoprotectant at −20°C before use. Mouse brains were dissected out after vascular perfusion with cold saline, with hemi-brains either snap-frozen for biochemical study or fixed by immersion in 4% paraformaldehyde for anatomical study.

### Immunohistochemistry and Immunofluorescence

Four sets of consecutive mid-hippocampal temporal lobe sections from each brain, 4–6 cases together in each experiment, were stained immunohistochemically with four antibodies: goat anti-sortilin extracellular domain (diluted at 1:2000, AF3154, immunogenic peptide corresponding to amino acid (a.a.) 76–753 of human sortilin, AF3154-SP, R&D Systems China Co. Ltd., Shanghai, China), rabbit anti-sortilin intracellular C-terminal domain (1:2000, ab16640, immunogenic peptide corresponding to a.a. 800–831 of human sortilin, ab16686, Abcam Trading Shanghai Company Ltd., Shanghai, China), monoclonal mouse anti-Aβ 6E10 (1:5000, #39320, Signet Laboratories Inc., Dedham, MA, USA) and rabbit anti-phosphorylated tau (1:5000, T6819, Sigma-Aldrich, St. Louis, MO, USA). In this set of experiments, the sections subjected to 6E10 labeling were invariably pretreated with formic acid for 1 h at room temperature. To determine the effect of several section pretreatments, including sortilin immunogenic peptide blocking (at 5 and 10 times of primary antibody concentration), heating (65°C), formic acid (100%) and guanidine hydrochloride (HCl; 5 M) treatments, consecutive sections from four brains with extensive amyloid pathology were selected for each treatment paradigm. Also, in each experiment, several adjacent sections were processed together with other sections, excluding the primary antibody in the incubation buffer, which were used to obtain the cutoff level of nonspecific labeling in densitometry.

Other than the above specifications, all sections were treated free-floating first with 5% H_2_O_2_ in PBS for 30 min and 5% normal horse serum in PBS with 0.3% Triton X-100 for 1 h, followed by incubation with the primary antibodies at 4°C overnight. The sections were then reacted with biotinylated horse anti-mouse, rabbit and goat IgGs at 1:400 for 1 h and ABC reagents (1:400; Vector Laboratories, Burlingame, CA, USA) for 1 h, with the immunoreactivity visualized in 0.003% H_2_O_2_ and 0.05% 3,3′-diaminobenzidine. Immunolabeling on paraffin sections was processed on-slide following dewaxing, rehydration and the antibody incubation steps described above, which involved Aβ, p-Tau, and sortilin immunolabeling with the rabbit antibody. The immunolabeled sections were used for the scoring of the AD type-pathology (i.e., ABC staging for amyloid pathology and Braak’s I–VI staging for tau pathology (Braak et al., 2006; Montine et al., 2012), and for an overall assessment for the presence of extracellular sortilin labeling relative to the occurrence of Aβ deposition.

Double immunofluorescence was initiated with section pretreatment in PBS containing 5% donkey serum for 30 min. The sections were then incubated overnight at 4°C with the rabbit sortilin antibody (ab16640, 1:1000), together with one of the following: (1) goat anti-sortilin (AF3154, 1:1000); (2) 6E10 (1:4000); (3) mouse anti-SYN (1:1000; MAB329, Millipore, MA, USA); (4) mouse anti-phosphorylated tau (PHF1, courtesy of Dr. P Davis, 1:4000); (5) mouse anti-glial fibrillary acidic protein (GFAP, 1:2000; MAB360, Millipore); and (6) mouse anti-ApoE (1:1000, NE1004, Millipore). Immunofluorescence was visualized with Alexa Fluor^®^ 488 and Alexa Fluor^®^ 594 conjugated donkey anti-mouse, anti-rabbit or anti-goat IgGs (1:200, Invitrogen, Carlsbad, CA, USA). The sections were treated with 0.1% Sudan black to block autofluorescence before microscopic examination.

### Western Blot

Preparation of tissue lysates and electrophoresis were carried out with caution to minimize the effect of temperature on protein degradation. Neocortex from the middle temporal gyrus of the frozen human cerebral hemispheres and frontal cortex from frozen mouse brains were sampled in the chamber of cryostat, then homogenized on ice by sonication in T-PER extraction buffer (Pierce, Rockford, IL, USA) containing protease inhibitors (Roche, Indianapolis, IN, USA). Resulting lysates were centrifuged at 15,000 *g* at 4°C, with supernatants collected and protein concentrations measured by DC protein assay (Bio-Rad Laboratories, Hercules, CA, USA). Extracts containing equal amount of total protein were run in SDS-polyacrylamide gel electrophoresis (PAGE) gels (protein loading and SDS concentration to be detailed along with result description). Separated protein products were electrotransferred to Trans-Blot pure nitrocellulose membranes, which were immunoblotted with the aforementioned antibodies (rabbit and goat anti-sortilin diluted at 1:2000, 6E10 at 1:4000, BACE1 at 1:2000 and phosphorylated tau at 1:2000; Cai et al., 2010), and that for loading controls including β-tubulin-III (1:5000, Millipore), β-actin (1:5000, Millipore) or glyceraldehyde-3-phosphate dehydrogenase (GAPDH; 1:5000, Millipore). The membranes were further reacted with HRP-conjugated secondary antibodies (1:20,000; Bio-Rad Laboratories). Immunoblotting signal was visualized with the ECL-Plus detection kit, followed by X-ray film exposure and image capture in a laser scanner.

### Imaging, Data Analysis and Figure Preparation

Immunolabeled sections were examined on an Olympus BX51 microscope and a Nikon confocal microscope equipped with digital imaging systems (CellSens Standard and Nikon-EZ-C1, respectively). Light microscopic images were taken using 2× to 40× objectives, with low magnification images montaged to re-construct the labeling over the temporal lobe regions. Confocal images were taken at 20× and 40× through three scans covering ~6 μm tissue depth. Images prepared for densitometry were obtained from comparable locations in adjacent sections using the same light intensity and digital exposure pre-settings, for all cases and all types of immunolabeling. Quantification of immunolabeled profiles was carried out following incorporation of region-matched images into a single file, which was converted into gray-scale TIFF format. Subsequently, either the areas or optic densities of the profiles/anatomic regions of interest were measured with the OptiQuant software (Packard Instruments, Meriden, CT, USA). Optic densities over immunoblotted protein bands were obtained with the same software.

All image data were exported into Excel spreadsheets and re-arranged according to groups, with specific densities calculated by background subtraction, and areal fractions or relative density levels calculated against internal references, whenever applicable. Finally, all data were entered into Prism spreadsheets, analyzed statistically, and graphed if applicable (GraphPad Prism 4.1, San Diego, CA, USA). Statistical analyses were conducted using either paired two-tailed *t*-test or a nonparametric test (Kruskal–Wallis with Dunn’s multiple comparison) with the GraphPad Prism software. Details regarding group pairing, sample size and statistics for individual tests will be incorporated in the following sections along with result description as well as in figure legend. The minimal significant level of difference was set at *p* < 0.05. Figures were assembled with Photoshop 7.1.

## Results

### Antibodies Against Sortilin Extracellular and Intracellular Domains Displayed Identical Neuronal Labeling in Adult Rodent and Human Cerebrum

**Figure 1:**
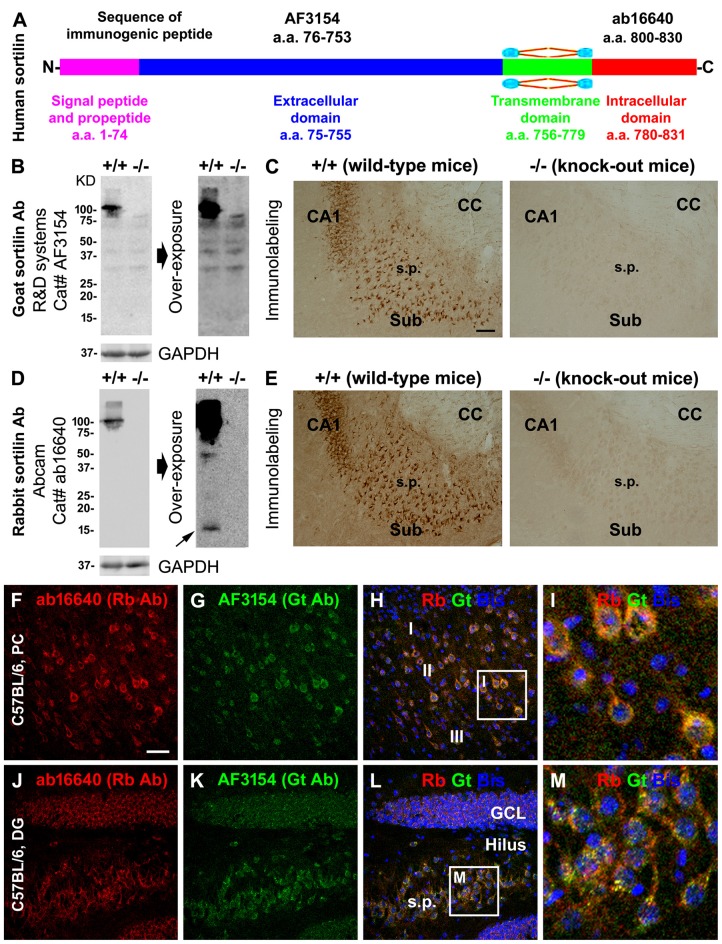
Validation of sortilin antibodies using sortilin knockout (−/−) and wildtype (+/+) mouse brains, with a characterization of the normal expression pattern of sortilin in rodent cerebrum. Panel **(A)** is a schematic drawing of the human sortilin protein, with the extracellular, transmembrane and intracellular domains of varying lengths of amino acid (a.a.) residues as marked. The immunogenic synthetic peptide sequences for the extracellular and intracellular C-terminal domain antibodies are also provided. Panels **(B,C)** show representative immunoblot and immunolabeling results obtained with the goat antibody (Gt Ab) against the extracellular domain in sortilin +/+ and −/− brains. Panels **(D,E)** show the results obtained with the rabbit antibody (Rt Ab) against the intracellular C-terminal. Both antibodies label a ~100 kDa band in sortilin +/+, but not in −/−, lysates **(B,D)**. Several non-specific bands are visible in the immunoblot with the goat antibody, equally present in the sortilin +/+ and −/− lysates, by extending the time of film exposure **(B)**. With overexposure, two light bands at ~40 and ~15 kDa (pointed by arrow) are also noticeable in the immunoblot of sortilin +/+ lysates with the rabbit antibody **(D)**. Light microscopic images **(C,E)** show neuronal profiles in the subiculum (Sub) to CA1 transitional region labeled by both antibodies in sortilin +/+, but not in −/−, brain sections. Confocal immunofluorescent images show completely colocalized labeling by the two antibodies in cortical pyramidal-like neurons **(F–I)**, CA3 pyramidal neurons and granule cells of the dentate gyrus (DG) **(J–M)** in C57BL mouse brain, with granular elements seen intracellularly **(I,M)**. Western blot applications: 12% SDS-PAGE gel and 13 μg equal amount protein loading. Additional abbreviations: CC, corpus callosum; s.p., stratum pyramidale; GCL, granule cell layer; PC, Parietal cortex; I-III, cortical layers; GAPHD, glyceraldehyde-3-phosphate dehydrogenase. Scale bar = 200 μm in **(C)** applying to **(E)**; 100 μm in **(F)** applying to **(G,H,J–L)**, equivalent to 25 μm for **(I,M)**.

We validated the specificity of the sortilin antibodies to the extracellular (goat) and intracellular C-terminal (rabbit) parts using sortilin knockout (−/−) and wildtype (+/+) mouse brains (Figures 1A–E; Ruan et al., 2016). Both antibodies marked a ~100 kDa band in immunoblot of sortilin+/+, but not −/−, lysates, representing a presence of the full-length protein in the wild-type but not knockout mouse tissues (Figures 1B,D). By extending the film exposure time, two bands at ~15 kDa and ~40 kDa became visible in the immunoblot with the C-terminal antibody of the sortilin+/+, but not sortilin−/−, lysates (Figure 1D), indicating the existence of specific sortilin-derived peptides in the former preparation at very low concentration. In immunohistochemistry, the two antibodies exhibited identical, essentially neuronal, labeling in the sortilin+/+ mouse forebrain (Figures 1C,E), whereas the labeling appeared background like in sortilin−/− counterpart (Figures 1B,D). We also confirmed a fully co-localized immunofluorescent labeling by the two antibodies, occurring primarily in the somata and dendrites of cortical and hippocampal pyramidal neurons as well as the granule cells of dentate gyrus (DG) in wild-type rodents, including at granular profiles inside the somata and large dendrites (Figures 1F–M).

**Figure 2:**
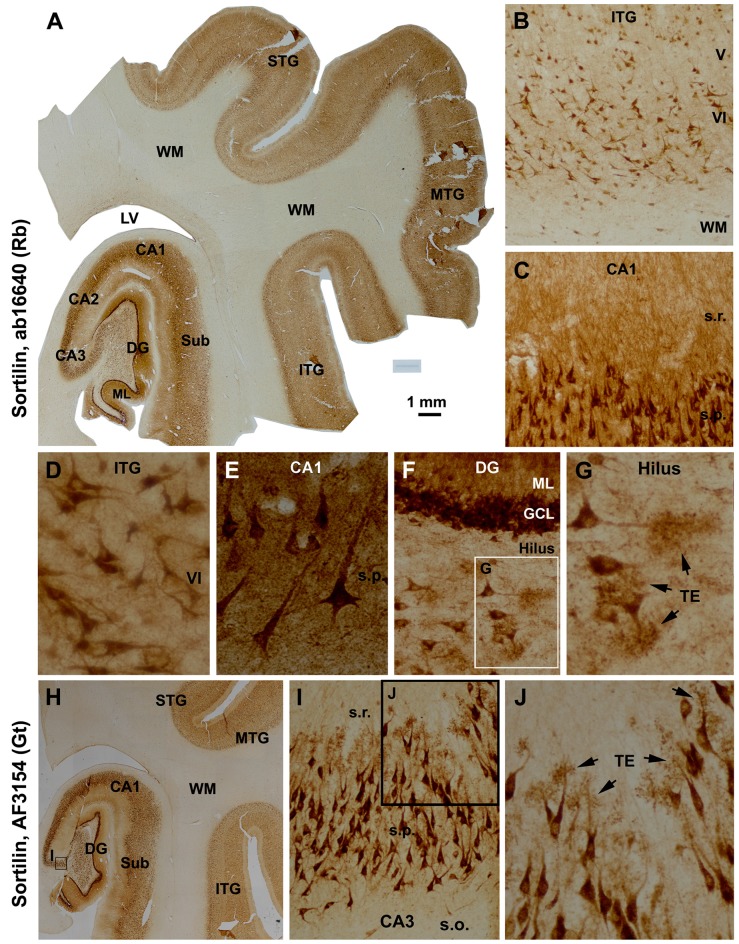
Characterization of the normal sortilin expression pattern in the human cerebral neocortex and hippocampal formation with tissue from a mid-age case. Panel **(A)** is a montaged low magnification image illustrating the expression of sortilin labeled with the rabbit antibody across the superior, middle and inferior temporal gyri (STG, MTG, ITG), subiculum (Sub), hippocampal Ammon’s horn (CA1–3) and DG. The labeling occurs in the gray matter but barely in the white matter (WM). The molecular layer (ML) of the DG shows strong neuropil reactivity. Panels **(B,D)** show higher power views of the labeling in the neocortex, with large and middle-sized pyramidal and polymorphic neurons exhibiting strong immunoreactivity. Panels **(C,E)** show heavy labeling in CA1 pyramidal neurons specifically in the somata and dendrites, with the stratum radiatum (s.r.) also exhibiting strong reactivity in fine dendritic processes and neuropil. Panels **(F,G)** are higher magnification images of the DG; the somata and proximal dendrites of the granule cells are intensively stained. The somata and dendrites of hilar mossy cells are distinctly labeled, with the thorny excrescences (TE, arrows) well-displayed. Axons of the cortical and CA1 pyramidal neurons are not visible **(D,E)**, with no labeling seen in the mossy fiber terminal field **(A,F)**. Panel **(H)** shows that the goat antibody exhibits essentially the same neuronal labeling pattern in the hippocampal formation and temporal neocortex as with the rabbit antibody **(A)**, with enlarged views specifically illustrating the visualization of the TE on the dendrites of the CA3 pyramidal neurons **(I,J)**. Additional abbreviations: IV-VI, cortical layers. Scale bar = 1 mm in **(A)**, equivalent to 2 mm for **(H)**, 200 μm for **(B)**, 100 μm for **(C,I)**, 50 μm for **(D,F,J)** and 25 μm for **(E,G)**.

The two sortilin antibodies also exhibited identical labeling pattern in mid-age human cerebrum (Figure 2). Thus, immunolabeling in the neocortex occurred in the gray matter, mainly over layers II–VI and heaviest in layers VI, with little reactivity in the WM (Figures 2A,H). At higher magnifications, pyramidal and polymorphic neurons in layers V/VI and II/III were among the most distinctly labeled (Figures 2A,B,D). The somata and apical dendrites of the subicular and hippocampal pyramidal neurons were clearly visualized, with granular elements visible in the cytoplasm (Figures 2C,E). In the DG, both the granule cell layer (GCL) and molecular layer (ML) showed heavy reactivity (Figures 2A,F). Hilar mossy cells and CA3 pyramidal neurons, including their dendritic trees, were well-labeled (Figures 2A,F,G,H,J), with the thorny excrescences (TE) on the dendrites clearly identifiable at high magnifications (Figures 2F,G,I,J). Overall, axonal profiles were not identifiable around the somata of cortical and hippocampal neurons (Figures 2D,E), nor in the cerebral WM (Figures 2A,H) and in the mossy fiber terminal field (Figures 2A,F,H).

### Sortilin C-Terminal Antibody Labeled Plaque-Like Lesions in Aged and AD Human Cerebrum

**Figure 3:**
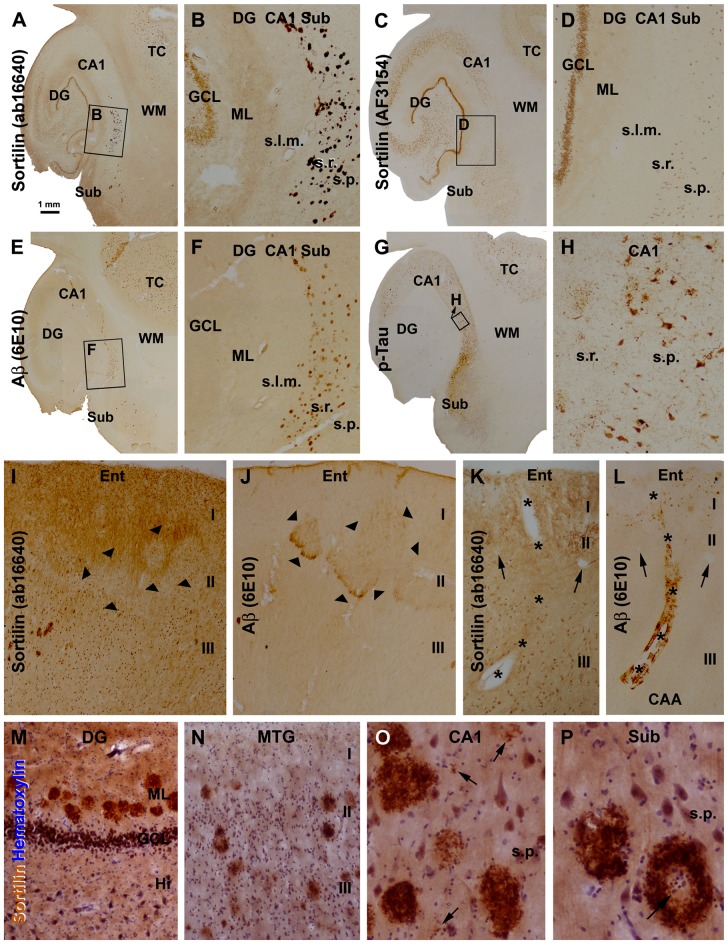
Morphological characterization of sortilin labeled plaque lesions relative to amyloid and tau pathology using sections from cases with Alzheimer’s disease (AD). Panels **(A–G)** show low and high power (framed areas) views over the hippocampal formation and part of temporal cortex (TC) from adjacent sections, with the 4 markers as indicated. The rabbit sortilin antibody labels plaque lesions that are regionally matched to that labeled by the 6E10 β-amyloid (Aβ) antibody **(A,B,E,F)**. Both the rabbit and goat sortilin antibodies label neuronal profiles seen clearly in the GCL at low magnification (a-d). Immunolabeling of phosphorylated Tau (p-Tau) is seen in tangled neurons and neuritic plaques **(G,H)**. Panels **(I,J)** are images taken from adjacent sections of the entorhinal cortex (Ent) from another AD case, showing a lack of extracellular sortilin labeling at the layer II cell islands (pointed by arrowheads) that exhibited diffuse Aβ deposition. Panels **(K,L)** shows a lack of sortilin deposition in association with cerebral amyloid angiopathy (CAA), also as assessed between consecutive sections. Asterisks denote the trajectory of a longitudinally cut amyloid arteriole entering the cortex from pia surface, with the two arrowheads pointing to two cross-sectioned normal vessels, for additional spatial reference. Panels **(M–P)** show high magnification views of extracellular sortilin deposits in sections counterstained with hematoxylin. Plaques occur in the ML of the DG in a row **(M)**, and in the cortex of the middle temporal gyrus (MTG) with different sizes and labeling intensity **(N)**. Panel **(O)** shows the occurrence of small amount of deposits (pointed by arrows) in the neuropil in areas about the sizes of the cell nuclei and neuronal somata, along with densely packed mature-looking plaques. Panel **(P)** shows circular plaques with cell nuclei (arrows) in the center. Additional abbreviations: I-III, cortical layers; s.l.m., stratum lacunosum-moleculare; other abbreviations are as defined in Figure 1. Scale bar = 1 mm in **(A)** applying to **(C,E,G)**; equivalent to 200 μm for **(B,D,F,I,J)**, 50 μm for **(K–N)** and 25 μm for **(O,P)**.

The sortilin antibodies against the extracellular and intracellular domains exhibited sharply different labeling patterns in the brains with AD-type pathology. Thus, the C-terminal antibody visualized plaque-like lesions, whereas the extracellular domain antibody did not (Figures 3A–H; Supplementary Figures S1, S2). Notably, in the samples with extensive plaque lesions, the neuronal labeling by the C-terminal antibody became less impressive, compared to that by the extracellular domain antibody (Figures 3A–D; Supplementary Figures S1, S2). This phenomenon appeared to likely reflect a competitive situation among the antigen epitopes in the section to bind the available antibody, because we found that the neuronal labeling could be enhanced by raising the concentration (from 1:2000 to 1:1000 or 1:500) of the rabbit antibody in the incubation buffer (data not shown).

We carried out an overall assessment of the C-terminal antibody labeling in sections from more than 70 brains with and without amyloid and tau pathology (including 10 Caucasian AD cases; Supplementary Table S1). Overall, the plaque-like sortilin labeling was found consistently among the cases that also had cerebral amyloid pathology. Thus, as assessed between consecutive temporal lobe sections, sortilin and Aβ labeled plaques both occurred over the neocortex, subiculum and hippocampal areas with their distributions matchable in reference to given subregions and lamina, noticeable at low magnification (Figures 3A,B,E,F; Supplementary Figures S1, S2). As with Aβ plaques, sortilin plaques lacked a spatial parallelism with tau pathology over the temporal lobe regions (Figures 3A–H; Supplementary Figures S1, S2) (therefore we did not quantitatively analyze sortilin relative to tau pathology). While assessing the overall pattern of extracellular sortilin/Aβ labeling, it should be notified that, along with the compact-like lesions diffuse Aβ deposition was present in local areas of the cortex, e.g., around layer I, the cell islands in layer II of the entorhinal cortex and the WM. In comparison, immunoreactivity of the C-terminal sortilin antibody in the above anatomic locations appeared to be not changed (relative to adjacent regions; Figures 3I,J; Supplementary Figures S2F,I). Also as a general note, 6E10 was found to label β-amyloidosis locally around the pia and blood vessels in the gray and WMs, whereat no increased extracellular sortilin deposition was found at these locations (Figures 3K,L).

At high magnifications more clearly in immunolabeled sections with hematoxylin counterstain, the plaque-like lesions detected with the sortilin C-terminal antibody consisted of extracellularly deposited fibrillary products (Figures 3M–P). The plaques were largely round, varying in size, often with the fibrillary material densely packed across the entire plaque. However, some plaques contained less densely stained fibrils (Figure 3N). Coexisting with the above, labeled extracellular fibrils were seen to deposit over small areas about the sizes of the neuronal nuclei, or as mini-plaques of the sizes of the neuronal perikarya (Figure 3O). Large sortilin plaque profiles could be ring-like with the deposits packed on the periphery, leaving an empty or pale center that sometimes occupied by cell nuclei (Figure 3P).

**Figure 4:**
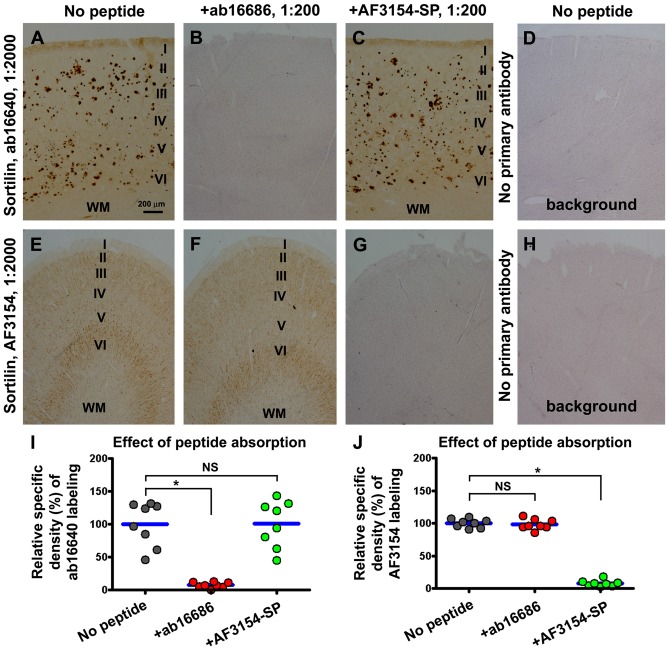
Verification of the specific labeling of the two sortilin antibodies in neuronal and plaque profiles with immunogenic peptide absorption assay. Panels **(A–C)** show representative images from sections processed with the rabbit antibody in the absence **(A)** and presence of the C-terminal **(B)** or the extracellular domain **(C)** immunogenic peptide at 10 times of the concentration of the primary antibody. Panel **(D)** shows the background reactivity seen in the cortex from the sections processed by excluding the primary antibody. Panels **(E–H)** show the labeling with the goat antibody arranged as indicated. Dot graphs **(I,J)** represent the relative specific optic densities obtained from a set of precentral and a set of postcentral gyral sections, from each of four brains with stage C amyloid pathology. The specific density is defined by subtracting the background from the total densities measured over layers I–VI of the same gyral region for each set of preparations, with the background density obtained from the section processed by excluding the primary antibody (without hematoxylin counterstain). Data from individual sections (i.e., totally eight sections) are normalized to the mean of the group with sections immunolabeled with the primary antibody only. To illuminate the cortical lamination, the sections used to prepare the images shown in panels **(B,D,G,H)** have been lightly counterstained with hematoxylin. The C-terminal and extracellular domain immunogenic peptides can selectively and completely block the immunolabeling of the corresponding sortilin antibodies **(I,J)**. Results from statistical analysis (Kruskal-Wallis analysis with *post hoc* test) between the indicated groups are as marked, with *noting *P* < 0.05 and NS noting not significant different **(I,J)**. Scale bar = 200 μm in **(A)** applying to other image panels.

We used immunogenic peptide absorption to verify the specific labeling of the sortilin antibodies in the human brain sections. The labeling of the rabbit antibody was diminished to the background levels (defined using sections processed in the absence of primary antibody), when the immunohistochemistry was proceeded by co-incubation with the C-terminal immunogenic peptide at either 5 (data not shown) and 10 times of the concentration of the primary antibody (Figures 4A,B,D,I). Such an effect did not occur by including the extracellular domain peptide in the incubation buffer (Figures 4A,C,I). Vice versa, the extracellular immunogenic peptide could completely block the labeling of the goat antibody, but did not affect the immunolabeling of the rabbit C-terminal antibody (Figures 4E–H,J).

### Sortilin and Aβ Deposition at Senile Plaques were Anatomically Correlated in Human Cerebrum

**Figure 5:**
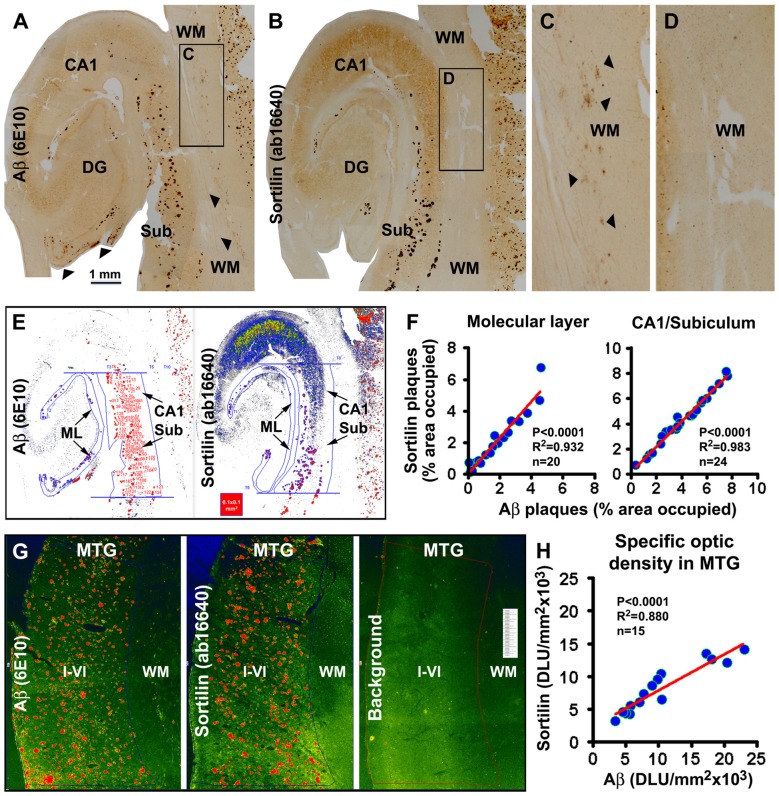
Quantitative analysis of sortilin relative to β-amyloid deposition in hippocampal lamina and temporal neocortex. Panels **(A,B)** show labeling with the rabbit sortilin antibody and 6E10 in consecutive temporal lobe sections used for quantification. The framed WM areas are enlarged to show diffuse Aβ labeling (pointed by arrows, also in panel **(A)** along the pia) that is not associated with extracellular sortilin deposition **(C,D)**. The pseudocolored screen print image **(E)** illustrates the methodology to measure the fractional areas of sortilin and Aβ plaques manually over the ML and in the CA1/subiculum transitional region. The latter is defined by two parallel lines passing the upper and lower edges of the GCL and between the hippocampal fissure and gray/WM border. Areas of individual plaques are measured by tracing along their outer border using the irregular selecting line-drawing tool (OptiQuant software). The sum of plaque-occupied areas is divided by the total area of the reference region, yielding the fractional areal value, expressed as % of area occupied, for each case. The fractional areal values for sortilin and Aβ plaques are plotted against individual aged and AD cases, with sample size noted in the graphs. In both the ML and the CA1/subiculum transitional region, the fractional areas of sortilin and Aβ plaques are positively correlated among the cases **(F)**. Pseudocolored image **(G)** illustrates the methodology to obtain the total densities of sortilin and 6E10 labeling over the same cortical area (layers I–VI) of the middle temporal gyrus (MTG) in consecutive sections, and to obtain the background cutoff density from another adjacent section batch-processed excluding primary antibodies. Specific optic density is calculated by subtracting the background from total densities. Panel **(H)** plots specific densities of sortilin and 6E10 immunolabeling relative to individual cases analyzed, showing a positive correlation between the two measurements. Statistical results obtained via a nonparametric (Kruskal-Wallis) test are also marked in the graphs **(F,H)**. Scale bar = 1 mm in **(A)** applying to **(B)**, equal to 250 μm for **(C,D)**.

In a subset of the aged and AD cases we noticed sortilin/Aβ plaques preferentially distributed along the ML of the DG, with distinctly labeled plaques also present in the subiculum and adjoining CA1 area (Figures 3K, 5). Therefore, we carried out correlative areal ratio analyses of sortilin and Aβ plaques across the ML and in a defined area of the CA1/subiculum region. The fractional areas of the sortilin and Aβ plaques, expressed as % area occupied, were plotted relative to individual cases (Figures 5A,B,E). A strong positive correlation (*P* < 0.0001, *R*^2^ = 0.932) between the fractional areas of sortilin and Aβ plaques in the ML was found based on the analysis of sections from 20 individuals (Figure 5F). In the CA1/subiculum transitional region selected for quantification, the fractional areas of sortilin and Aβ plaques were also positively correlated (*P* < 0.0001, *R*^2^ = 0.983, *n* = 24) in a case-dependent manner (Figures 5E,F). We further assessed if the overall amounts of sortilin and Aβ deposition were correlated in the neocortex, using a densitometric approach (Figure 5G). The resulting densitometric data indicated a positive correlation (*P* < 0.0001, *R*^2^ = 0.880, *n* = 15) between sortilin and Aβ deposition in reference to individual brains (Figure 5H). It should be noted that the total optic density measured in a region reflected that contributed by labeled plaques as well as neurons. In the case of Aβ immunolabeling, diffuse plaques and vascular β-amyloidosis present in the measured areas would also contribute to the yielded total optic density. Nonetheless, the densitometric correlation reflected a trend of overall parallelism between sortilin and Aβ extracellular deposition in the neocortex.

**Figure 6:**
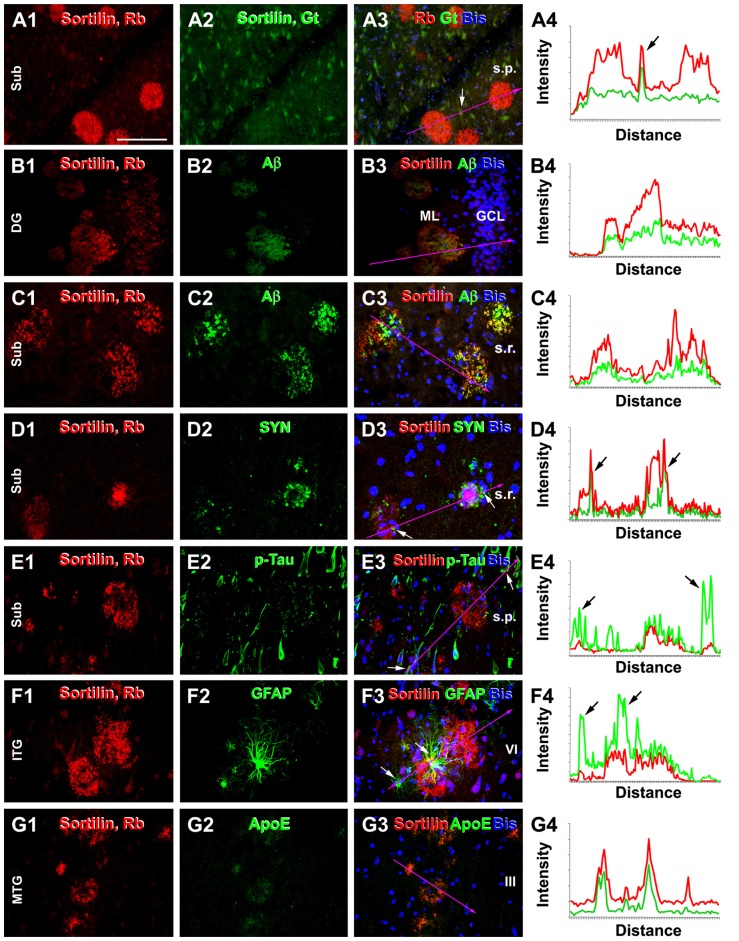
Double immunofluorescent characterization of sortilin labeling at neuritic plaques. Each set shows the immunolabeling with the rabbit sortilin antibody relative to another marker, with bisbenzimide nuclear labeling included in the merged image. Imaged region and lamina are as indicated. Histograms illustrate the relative intensity of the red and green fluorescence measured along the line marked in the fluorescent images, with arrows pointing to the locations of labeled cellular or neuritic elements. Panels **(A1–4)** show a selective labeling by the rabbit sortilin antibody at plaques, while neuronal somata are labeled by both sortilin antibodies. Panels **(B1–4)** show sortilin and Aβ labeling with parallel intensity among plaques in the ML. Panels **(C1–4)** show an overall colocalization of sortilin with Aβ in the plaques, while the fluorescent intensity is not parallel at local areas between the two markers (merged color appears more greenish, reddish or neutrally yellow). Panels **(D1–4)** show sortilin labeling coexisting but not colocalizing with dystrophic neurites (DNs) with distinct synaptophysin (SYN) immunoreactivity at the same plaques. Panels **(E1–4)** show extracellular sortilin labeling coexisting with DNs with weak phosphorylated-Tau (p-Tau) reactivity. Panels **(F1–4)** show sortilin plaques surrounded by hypertrophic astrocytes with bright GFAP immunofluorescence. Panels **(G1–4)** display colocalization of extracellular sortilin and ApoE labeling at plaques. Scale = 100 μm in **(A)** applying to all image panels.

In double immunofluorescence, only the rabbit antibody, but not the goat antibody, labeled plaques, while there was a colocalization of the labeling by the two antibodies in neuronal somata (Figures 6A1–4). Colocalization of extracellular sortilin and Aβ labeling at individual compact-like plaques was clearly seen in the hippocampal and cortical regions, shown for examples in the ML of the DG (Figures 6B1–4) and the subiculum (Figures 6C1–4). Notably, while the immunofluorescence appeared to be parallel in intensity between the two markers across the entire area of a plaque (Figures 6B1–4); it could be stronger for one marker than the other over subareas of the same plaque (Figures 6C1–4). Sortilin and SYN immunofluorescence co-existed at apparently neuritic plaques, with SYN-labeled DNs surrounded by or intermingled with extracellular sortilin labeling (Figures 6D1–4). Extracellular sortilin labeling was also found to coexist with neuritic elements with weak p-Tau immunofluorescence in plaques (Figures 6E1–4). Hypertrophic astrocytes with bright GFAP immunofluorescence were present in the vicinity of some sortilin labeled plaques (Figures 6F1–4). Moreover, sortilin-labeled plaques colocalized with extracellular ApoE labeling (Figures 6G1–4).

### Aβ-Enhancing Antigen Retrieval did not Affect Extracellular Sortilin Labeling

Tissue processing and antigen retrieval, e.g., temperature, formic acid and guanidine HCl, could affect Aβ aggregation or Aβ immunodetection. We therefore explored if sortilin labeling could be affected by some Aβ-enhancing antigen retrieval methods. These experiments were carried out by batch-processing consecutive frontal (blocked from the precentral gyrus) and parietal (postcentral gyrus) neocortical sections subjected to untreated and pretreated conditions. The sections from four brains with stage C Aβ pathology were used, with densitometric data obtained from the same gyral region.

**Figure 7:**
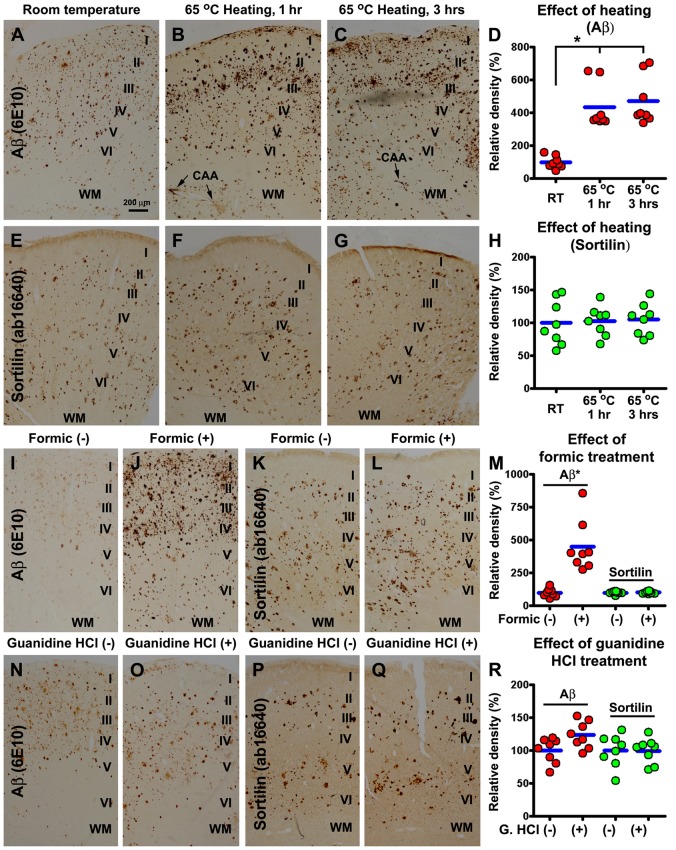
Comparative characterization of the effect of antigen retrieval pretreatments on sortilin and Aβ labeling using neocortical sections from cases with severe cerebral amyloid pathology. Adjacent sections of the precentral and postcentral gyri from four cases are first subjected to an antigen retrieval condition, with the treated and untreated sections subsequently processed together for immunohistochemistry. Optic densities measured from the same gyrus are normalized to the mean calculated based on the densities obtained from the untreated sections, and plotted in reference to individual sections (totally eight sections). Aβ immunoreactivity in sections preheated at 65°C for 1 h and 3 h is increased in overall intensity, and specifically in layer I, WM and vascular sites (i.e., cerebral amyloid angiopathy, CAA), relative to untreated (room temperature) control, significantly between the treated and untreated groups but not between the 1 h and 3 h groups by Kruskal-Wallis analysis with *post hoc* test **(A–D)**. On the contrary, sortilin immunoreactivity is not altered in the preheated relative to untreated sections **(E–H)**. Formic acid (100%) treatment for 1 h also greatly enhances Aβ labeling, but has little effect on sortilin labeling **(I–M)** (paired *t*-test). Guanidine Hydrochloride (HCl; 5 M) pretreatment for 1 h slightly increases Aβ labeling not reached significance (paired *t*-test), and it has no effect on sortilin labeling related to untreated control **(N–R)**. Cortical layers are marked in the image panels. *p < 0.05. Scale bar = 100 μm in **(A)** applying to all image panels.

Compared to control, sections preheated at 65°C for 1 h and 3 h had more intense Aβ immunolabeling, especially along the pia, over layer I, in the WM and at individual sites of cerebral vasculature (i.e., cerebral amyloid angiopathy, CAA; Figures 7A–C). The mean density of Aβ labeling measured over layers I–VI increased to 433.4 ± 134.3% and 470.9 ± 146.2% in the sections preheated for 1 h and 3 h, respectively, relative to control (100 ± 37.3%), exhibiting an overall difference among the three groups (*P* = 0.0003, KWI = 16.1), with significant difference between the control and both preheated groups, but not between the groups heated for 1 h and 3 h, by *post hoc* test (Figure 7D). On the contrary, section preheating did not alter the pattern and intensity of sortilin labeling (Figures 7E–H).

Section pretreatment with formic acid for 1 h and 3 h also greatly enhanced Aβ labeling relative to untreated controls, with the effect readily maximized by 1 h treatment therefore presented below. Quantitatively, the mean density of Aβ labeling in formic treated sections increased significantly (*P* < 0.0005, paired *t*-test), to over 4-fold (433.4 ± 134.3%) relative to untreated sections (100 ± 32.2%; Figures 7I,J,M). Again, formic acid treatment did not affect the distribution or intensity of sortilin labeling (Figures 7K–M). Pretreatment of sections with guanidine HCl for either 1 h or 3 h (not shown) appeared to enhance Aβ labeling slightly (however, not reached significance), relative to untreated control (Figures 7N,O,R). The distribution or the overall amount of sortilin labeling was not different between the treated and untreated sections (Figures 7P–R).

### Sortilin Fragments were Elevated in AD Relative to Control Human Cortical Lysates

**Figure 8:**
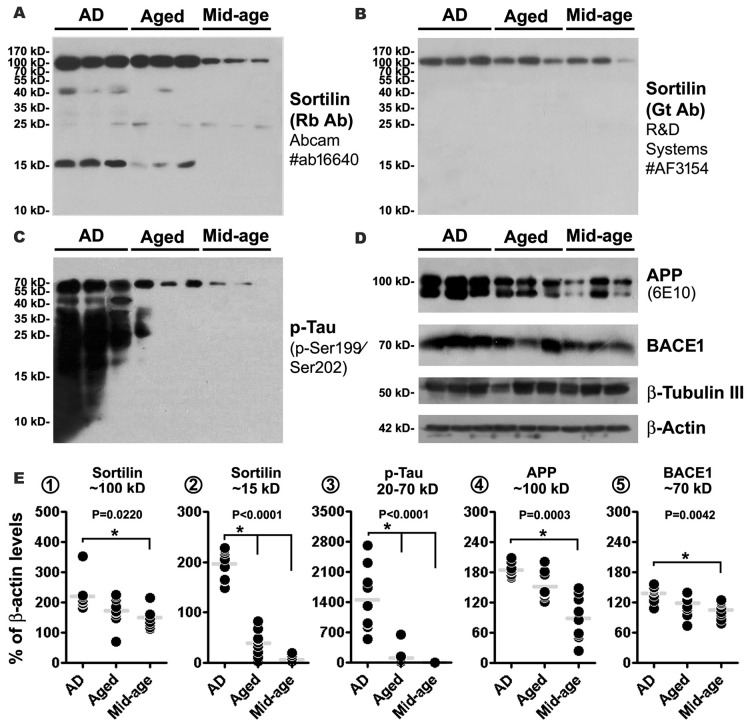
Western blot characterization of sortilin products in AD relative to aged and mid-age human temporal neocortical lysates. Samples in the AD group are from cases (*n* = 9) with pathologically confirmed amyloid deposition in the contralateral hemisphere, scored ≥ B. Samples in the Aged and Mid-age groups are from cases without microscopically evident amyloidosis. Panels **(A–D)** illustrate a set of representative images (three cases per group) of immunoblots assayed with sortilin and AD related markers, as indicated. Panels **(E1–5)** show the quantification and statistical result (Kruskal-Wallis non-parametric test with *post hoc* test, same for below) for each marker based on data from nine cases of each group. The rabbit and goat antibodies detect the same ~100 kDa band representing the full-length sortilin protein, with its median density elevated in the AD relative to Mid-age groups **(A,B,E1)**. The rabbit antibody additionally detects a major fragment at ~15 kDa, with its levels elevated in the AD group relative to Aged and Mid-age groups **(A,E2)**. Immunoblotted phosphorylated tau (p-Tau) products appear as a smear of bands less than 70 kDa, with the levels measured for 20–70 kDa bands dramatically elevated in the AD relative to Aged and Mid-age groups **(C,E3)**. Levels of amyloid precursor protein (APP) and β-secretase-1 (BACE1) are also elevated in the AD as compared to the Mid-age group **(D,E4,5)**. Western blot application: 15% SDS-PAGE gel and 35 μg equal protein loading for sortilin and p-Tau antibodies; 10% SDS-PAGE gel and 35 μg equal protein loading for other antibodies. P value for the overall difference between groups is indicated for each marker, with statistically significant inter-group difference (*P < 0.05) by *post hoc* also marked in the graph.

Western blot was used to explore the putative sortilin products found deposited at neuritic plaques, with an attempt to identify potential dependent effect of the presence of amyloid pathology as well as brain aging. Thus, temporal neocortical samples (*n* = 9/group, from the frozen hemisphere) were obtained from the cases in which the fixed contralateral hemispheres were pathological examined (see Supplementary Table S1). Cortical lysates were immunoblotted with the two sortilin antibodies, and antibodies to APP, BACE1 and p-Tau (as pathological controls). The rabbit sortilin antibody blotted the principal band migrated at ~100 kDa, which appeared thicker in the lysates from the AD and Aged relative to Mid-age groups. Another prominent band blotted by this antibody migrated at ~15 kDa, which was present in lysates from the AD and Age groups, denser in the former, while it was absent or minimal in the lysates from Mid-age subjects (Figure 8A). The medians of normalized (to β-actin signal) densities of the 100 kDa band were significantly different in the AD (mean ± SD = 209.6 ± 9.4%, same format below), Aged (192.2 ± 29.3%) and Mid-age (147.2 ± 38.8%) groups (*P* = 0.0220, Kruskal-Wallis statistic index, i.e., KWI, 7.6), with Dunn’s Multiple Comparison Test reported a statistical difference between the AD and Mid-age groups (Figure 8E1). The median densities of the 15 kDa product were different in the three groups (*P* < 0.0001, KWI = 21.3), with *post hoc* test indicating a significant increase in the AD (190.2 ± 34.7%) relative to Aged (44.9 ± 27.1%) and Mid-age (6.8 ± 7.7%) groups (Figure 8E2). A light ~40 kDa band was also visualized by the rabbit antibody in some AD and Aged cases, but not in the Mid-age group (Figure 8A) (not quantified as it was not consistently present). Different from the rabbit antibody, the goat sortilin antibody only blotted the ~100 kDa band (Figure 8B, quantitative data not shown), which showed a parallel trend in density between the cases as that blotted by the rabbit antibody. Immunoblotted p-Tau products occurred in large amounts in the lysates from the AD cases, appearing as a smear of bands ≤70 kDa (Figure 8C). The median density of the p-Tau products was increased in the AD group (1455.1 ± 745.0%) relative to Aged group (109.4 ± 205.5%) and Mid-age group (2.5 ± 3.8%) (*P* < 0.0001, KWI = 20.2) (Figure 8E3). The bands of full-length APP protein (~100 kDa) blotted with the 6E10 antibody appeared to be denser among the AD cases (Figure 7E), with an overall difference of its levels between AD (183.9 ± 13.2%), Aged (151.6 ± 27.4%) and Mid-age (88.1 ± 43.9%) groups (*P* = 0.0003, KWI = 16.0), elevated in the AD relative to the Mid-age groups by *post hoc* test (Figure 8E4). The amount of BACE1 protein (~70 kDa) appeared to be increased among the AD cases relative the other two groups (Figure 8D), with densitometry indicating an overall difference (*P* = 0.0003, KWI = 16.0) between the AD (138.7 ± 15.1%), and Aged (118.2 ± 22.1%) and Mid-age (105.9 ± 16.2%) groups, and significant difference between the AD and Mid-age groups by *post hoc* test (Figure 8E5). Amounts of β-actin (used as a total protein loading control) and β-tubulin (used as a neuronal protein control) appeared to be comparable between the samples (Figure 8D).

## Discussion

### Full-Length Sortilin Is Enriched in the Somatodendritic Compartment of Cerebral Neurons

Sortilin is a type I transmembrane protein consisted of 831 a.a. residues in Homo sapiens, with a long extracellular N-terminal sequence (755 a.a. in length), a single-pass transmembrane domain (a.a. 756–779) and a short intracellular C-terminal tail (a.a. 780–831) (Westergaard et al., 2004; Hermey, 2009). First cloned from human brain (Petersen et al., 1997), sortilin is also called as neurotensin receptor-3 for an initially identified role in neurotensin signaling (Mazella et al., 1998; Sarret et al., 2003). Follow-up cell biology studies indicate that it plays a general role in sorting/trafficking of membrane proteins between intracellular organelles (Strong et al., 2012; Mortensen et al., 2014; Kjolby et al., 2015). Sortilin is also known as an ApoE receptor, and may play a key role in lipoprotein metabolism and involve in some cardiovascular and metabolic disorders (Carlo, 2013; Coutinho et al., 2013; Kjolby et al., 2015). In the nervous system, sortilin is shown to regulate the signaling of nerve growth factors and progranulin, which may relate to disease mechanisms underlying some neurological and mental disorders (Nykjaer et al., 2004; Chen et al., 2005; Hu et al., 2010; Yang et al., 2011, 2013).

In this study, we have characterized the normal expression pattern of sortilin in adult human cerebrum with two antibodies targeting separately to the extracellular and intracellular domains. The specificity of the antibodies has been vigorously checked with three approaches, i.e., gene knock-out system, immunogenic peptide absorption and primary antibody omission. Since both antibodies essentially only label the ~100 kDa band in immunoblot of adult/normal rodent and human brain lysates, the immunolabeling seen microscopically would reflect the expression of full-length sortilin *in vivo*. Based on morphological characteristics, cortical and hippocampal principal neurons express high levels of the full-length protein, particularly in the somatodendritic compartment. The labeled granular elements seen in the somata and dendrites would represent intracellular organelles wherein protein sorting/trafficking takes place (Petersen et al., 1997; Nielsen et al., 2001; Gelling et al., 2012).

Often it is difficult with immunolabeling to determine whether a protein is localized to synaptic structures under the light microscope, especially in human brain samples obtained with postmortem delay. The finding of sortilin immunolabeling to the TE of hilar mossy cells and CA3 pyramidal neurons in normal human brain is remarkable. The visualization of this complex dendritic spine formation by both the extracellular and intracellular domain antibodies strongly suggests that the full-length sortilin protein is actually enriched at postsynaptic sites. Therefore, sortilin could play a physiological role in sorting/recycling of postsynaptic proteins, which could be important for synapse function and plasticity.

### Sortilin Fragments Are a Prominent Constituent of Senile Plaques in Human Cerebrum

Recent studies have explored sortilin in relevance to AD and age-related dementia at genomic, protein and cell biology levels. So far, no data have shown that sortilin gene mutation or polymorphic variation increases the risk of developing AD (Zeng et al., 2013). Notably, a late study reports that the SNP rs17646665 located in a non-coding region of *SORT1* gens is associated with a reduced risk for AD (Andersson et al., 2016). Three studies (to the best of our knowledge) have analyzed the 100 kDa sortilin protein in postmortem human brains, with its levels found either preserved in AD patients and subjects with mild cognitive impairment relative to people with normal cognition at death (Mufson et al., 2010), or elevated among AD relative to control elderly (Finan et al., 2011; Saadipour et al., 2013). Cell biology and proteomics studies suggest a regulatory interplay between sortilin and the amyloidogenic proteins. Thus, sortilin can mediate APP and BACE1 trafficking (Finan et al., 2011; Saadipour et al., 2013; Yang et al., 2013), while BACE1 and γ-secretase may catalyze sortilin proteolysis (Nyborg et al., 2006; Hemming et al., 2009).

In the present study, we show that a sortilin C-terminal antibody visualizes extracellularly deposited products in the human brains with cerebral amyloid pathology. The smallest amount of microscopically detectable deposits appears fibrillary, present in the neuropil covering areas about the sizes of neuronal nuclei and somata. The deposits appear to accumulate locally to form mini-plaques, dense-packing and ring-like mature-looking plaques. Neither the isolated deposits nor plaques occur preferentially surrounding the neuronal somata. Also, the deposits are not detectable inside neuronal somata and large dendrites. Quantitative analyses based on fractional areal measurement of individual plaques or collective densitometry of labeled profiles indicate a correlation between sortilin and Aβ deposition in the human cerebrum in a brain region/lamina- and case-dependent manner. Double immunofluorescent characterizations with a panel of markers co-existing at neuritic plaques, i.e., SYN, p-Tau, GFAP and ApoE (Kida et al., 1995; Zhang et al., 2009; Cai et al., 2010, 2012; Sadleir et al., 2016), show a colocalization of sortilin deposits in this type of amyloid plaques.

As elaborated in the “Introduction” Section, senile or neuritic plaques are historically referred to the silver stained lesions consisted of DNs and amorphous material, i.e., the amyloid product recognized later. In Aβ antibody stain, compact plaques in primitive, dense-packing or cored forms appear to closely represent neuritic plaques (while the so-called “burn-out” plaques may lack or contain only a small amount of DNs; Delaère et al., 1991). Aβ antibodies also label diffuse plaques as well as vascular and meningeal amyloidosis that often co-exist with compact plaques in the brain. Antigen retrieval methods, especially formic acid treatment, are routinely applied to Aβ immunohistochemistry. Diffuse plaques can show fairly dense labeling and mix with compact plaques in the same microscopic field. Thus, microscopic differentiation between the two types may not be always conclusive. In fact, inter-laboratory and inter-experimental inconsistency exists in the assessment of plaque lesions (Alafuzoff et al., 2008). Our comparative analyses on the effect of antigen retrieval indicate that section pretreatment with formic acid or heating essentially does not alter the amount and pattern of sortilin deposition, although dramatically enhances Aβ labeling and affects plaque appearance. Thus, the extracellular sortilin immunolabeling appears to be highly reproducible when used to visualize neuritic-like plaques in postmortem human brain.

Collectively, the data obtained from the human brains through comparative immunohistochemical and immunoblot assessments with the extracellular and intracellular domain antibodies allow us to conclude that the extracellularly deposited sortilin products must be the fragments rather than the parent protein. Specifically, the 15 kDa fragments detected only by the C-terminal antibody appear to be the major culprit behind the extracellular lesions. The levels of these fragments are dramatically elevated in the cortical lysates from AD human brains with amyloid pathology relative to aged/mid-age controls without microscopically overt amyloidosis.

### Sortilin Neuropathology Might Relate to Synaptic Degeneration

As an initially observed human pathology, many mechanistic questions await to address, such as how and why sortilin deposition occurs. Our current data allow some preliminary interrogation on a few issues, in reference to existing literature. The first one involves whether sortilin deposition occurs as a direct consequence of amyloidogenesis. While sortilin deposits coexist with Aβ at neuritic-like plaques in the gray matter, they are not evidently present along with meningeal and vascular β-amyloidosis or in areas with typical diffuse Aβ deposition in the gray and WMs. Accordingly, sortilin deposition could not be coherently explained as a passive event resulted from absorptive binding by Aβ. Neither sortilin full-length nor deposited products are labeled in the DNs. Therefore, it appears unlikely that the sortilin deposits derive from the DNs via secretase-mediated proteolysis of the parent protein. In comparison, increased BACE1 labeling can be found in DNs of neuritic plaques, swollen axons in the WM as well as in vascular and meningeal cells, implicating a potential role of Aβ overproduction by local cellular profiles for site-specific cerebral β-amyloidosis (Cheng et al., 2014; Deng et al., 2014; Yan et al., 2014; Xue et al., 2015; Devraj et al., 2016).

The second issue is about the anatomic origin of the sortilin deposits. Labeling of the full-length protein is localized primarily to neuronal profiles, enriched in the somatodendritic rather than axonal profiles (as discussed in the first part of “Discussion” Section). The giant spine formations, TE, are distinctly labeled. The labeling is also intense in the ML of the DG and the stratum radiatum of Ammon’s horn/subiculum likely in dendritic elements. Thus, dendritic branches and spines could be a major origin of the sortilin deposits. Such a notion fits well with the overall region-specific distribution of sortilin plaques in the neuropil of the gray matter, including the ML and stratum radiatum wherein neuronal somata are rare but plaques are distinct. Synaptic degeneration is considered the most important anatomical basis underlying cognitive decline in AD. Plaque formation appears to be a continuing process tightly associated with synaptic degeneration, with dendritic spine loss and axonal terminal degeneration/dystrophy at the lesion sites (Gonatas et al., 1967; Masliah et al., 1991; Ferrer et al., 1998; Adalbert and Coleman, 2013; Yan et al., 2014; Herms and Dorostkar, 2016; Sadleir et al., 2016). Sortilin fragments might derive largely from degenerating dendritic spines and branches therefore accumulate preferentially at neuritic-like plaques.

The third issue relates to the biochemical process that might underlie the formation of sortilin fragments. Some *in vitro* and *in vivo* data suggest that the extracellular domain of sortilin can shed off as soluble products (Navarro et al., 2002; Molgaard et al., 2016; Ogawa et al., 2016). The ~15 kDa products are exclusively immunoblotted by the C-terminal antibody, with the plaque labeling completely diminished by the corresponding blocking immunogenic peptide. With this information and in reference to the molecular weight (~15 kDa), the deposited sortilin products could be predicted to contain the C-terminal tail, transmembrane domain and a short part of the extracellular sequence (roughly a.a. 670–831 according to online protein analysis tools), without considering the situation of protein modification (the C-terminal region contains two putative phosphorylation sites). The sortilin fragments might be formed after the extracellular domain is cleaved off, and aberrantly accumulate site-specifically along with dendritic/spine degeneration of local neurons during plaque pathogenesis.

In summary, we have identified sortilin fragments as a new component of the abnormally deposited protein products in senile plaques in the human cerebrum. This sortilin extracellular pathology is fairly preferentially associated with neuritic-like amyloid plaques, but rarely occurs around the pia, at vascular wall and in regions with typical diffuse Aβ deposition. The plaque-like sortilin labeling is not affected by antigen retrieval methods that enhance Aβ immunoreactivity. The ~15 kDa fragments appear to be a major form of the deposited components, which is predicted to derive largely from the C-terminal region likely including the transmembrane part of the parent protein. Mechanistically, we speculate that these fragments are formed along with the degeneration of largely the dendritic spines and fine branches.

## Author Contributions

XH: immunohistochemistry and immunofluorescence; Z-LH and C-QL: western blot; ZL: confocal imaging; C-SR: antibody verification; W-YQ and CM: human brain banking and pathological characterization; AP, LS and B-ST: brain banking; YCai: imaging and data analysis; YChu: verification of the pathology in Caucasian AD brain samples; HC: data interpretation and article writing; X-FZ: antibody verification and data interpretation; X-XY: experimental design, human brain banking and pathological characterization, data analysis and interpretation; article writing.

## Conflict of Interest Statement

The authors declare that the research was conducted in the absence of any commercial or financial relationships that could be construed as a potential conflict of interest.

## References

[B1] Adalbert R. Coleman M. P. (2013). Review: axon pathology in age-related neurodegenerative disorders. Neuropathol. Appl. Neurobiol. 39, 90–108. 10.1111/j.1365-2990.2012.01308.x 23046254

[B2] Aisen P. S. (2005). The development of anti-amyloid therapy for Alzheimer’s disease: from secretase modulators to polymerisation inhibitors. CNS Drugs 19, 989–996. 10.2165/00023210-200519120-00002 16332141

[B3] Alafuzoff I. Pikkarainen M. Arzberger T. Thal D. R. Al-Sarraj S. Bell J.. (2008). Inter-laboratory comparison of neuropathological assessments of beta-amyloid protein: a study of the brainnet europe consortium. Acta Neuropathol. 115, 533–546. 10.1007/s00401-008-0358-2 18343933

[B4] Allsop D. Landon M. Kidd M. Lowe J. S. Reynolds G. P. Gardner A. (1986). Monoclonal antibodies raised against a subsequence of senile plaque core protein react with plaque cores, plaque periphery and cerebrovascular amyloid in Alzheimer’s disease. Neurosci. Lett. 68, 252–256. 10.1016/0304-3940(86)90152-7 3748453

[B5] Andersson C. H. Hansson O. Minthon L. Andreasen N. Blennow K. Zetterberg H.. (2016). A genetic variant of the sortilin 1 gene is associated with reduced risk of Alzheimer’s disease. J. Alzheimers Dis. 53, 1353–1363. 10.3233/jad-160319 27392867 PMC5147507

[B6] Andreasen N. Minthon L. Davidsson P. Vanmechelen E. Vanderstichele H. Winblad B.. (2001). Evaluation of CSF-tau and CSF-Aβ42 as diagnostic markers for Alzheimer disease in clinical practice. Arch. Neurol. 58, 373–379. 10.1001/archneur.58.3.373 11255440

[B7] Avci D. Lemberg M. K. (2015). Clipping or extracting: two ways to membrane protein degradation. Trends Cell Biol. 25, 611–622. 10.1016/j.tcb.2015.07.003 26410407

[B8] Borchelt D. R. Ratovitski T. van Lare J. Lee M. K. Gonzales V. Jenkins N. A.. (1997). Accelerated amyloid deposition in the brains of transgenic mice coexpressing mutant presenilin 1 and amyloid precursor proteins. Neuron 19, 939–945. 10.1016/s0896-6273(00)80974-5 9354339

[B9] Braak H. Alafuzoff I. Arzberger T. Kretzschmar H. Del Tredici K. (2006). Staging of Alzheimer disease-associated neurofibrillary pathology using paraffin sections and immunocytochemistry. Acta Neuropathol. 112, 389–404. 10.1007/s00401-006-0127-z 16906426 PMC3906709

[B10] Braak H. Braak E. (1991). Demonstration of amyloid deposits and neurofibrillary changes in whole brain sections. Brain Pathol. 1, 213–216. 10.1111/j.1750-3639.1991.tb00661.x 1669710

[B11] Burns M. P. Noble W. J. Olm V. Gaynor K. Casey E. LaFrancois J.. (2003). Co-localization of cholesterol, apolipoprotein E and fibrillar Aβ in amyloid plaques. Mol. Brain Res. 110, 119–125. 10.1016/s0169-328x(02)00647-2 12573540

[B12] Cai Y. Xiong K. Zhang X. M. Cai H. Luo X. G. Feng J. C.. (2010). β-secretase-1 elevation in aged monkey and Alzheimer’s disease human cerebral cortex occurs around the vasculature in partnership with multisystem axon terminal pathogenesis and β-amyloid accumulation. Eur. J. Neurosci. 32, 1223–1238. 10.1111/j.1460-9568.2010.07376.x 20726888 PMC2970759

[B13] Cai Y. Zhang X. M. Macklin L. N. Cai H. Luo X. G. Oddo S.. (2012). BACE1 elevation is involved in amyloid plaque development in the triple transgenic model of Alzheimer’s disease: differential Aβ antibody labeling of early-onset axon terminal pathology. Neurotox. Res. 21, 160–174. 10.1007/s12640-011-9256-9 21725719 PMC3227764

[B14] Carlo A. S. (2013). Sortilin, a novel APOE receptor implicated in Alzheimer disease. Prion 7, 378–382. 10.4161/pri.26746 24121631 PMC4134342

[B15] Chen Z. Y. Ieraci A. Teng H. Dall H. Meng C. X. Herrera D. G.. (2005). Sortilin controls intracellular sorting of brain-derived neurotrophic factor to the regulated secretory pathway. J. Neurosci. 25, 6156–6166. 10.1523/JNEUROSCI.1017-05.2005 15987945 PMC1201519

[B16] Cheng X. He P. Yao H. Dong Q. Li R. Shen Y. (2014). Occludin deficiency with BACE1 elevation in cerebral amyloid angiopathy. Neurology 82, 1707–1715. 10.1212/WNL.0000000000000403 24739782 PMC4032211

[B17] Coria F. Castaño E. Prelli F. Larrondo-Lillo M. van Duinen S. Shelanski M. L.. (1988). Isolation and characterization of amyloid P component from Alzheimer’s disease and other types of cerebral amyloidosis. Lab. Invest. 58, 454–458. 2965774

[B18] Coutinho M. F. Bourbon M. Prata M. J. Alves S. (2013). Sortilin and the risk of cardiovascular disease. Rev. Port. Cardiol. 32, 793–799. 10.1016/j.repc.2013.02.006 23910371

[B19] Cristóvão J. S. Santos R. Gomes C. M. (2016). Metals and neuronal metal binding proteins implicated in Alzheimer’s disease. Oxid. Med. Cell. Longev. 2016:9812178. 10.1155/2016/9812178 26881049 PMC4736980

[B20] Critchley M. (1929). Critical review: the nature and significance of senile plaques. J. Neurol. Psychopathol. 10, 124–139. 10.1136/jnnp.s1-10.38.124 21611296 PMC1038561

[B21] Delaère P. Duyckaerts C. He Y. Piette F. Hauw J. J. (1991). Subtypes and differential laminar distributions of Aβ4 deposits in Alzheimer’s disease: relationship with the intellectual status of 26 cases. Acta Neuropathol. 81, 328–335. 10.1007/bf00305876 1711758

[B22] Deng X. Li M. Ai W. He L. Lu D. Patrylo P. R.. (2014). Lipolysaccharide-induced neuroinflammation is associated with Alzheimer-like amyloidogenic axonal pathology and dendritic degeneration in rats. Adv. Alzheimer Dis. 3, 78–93. 10.4236/aad.2014.32009 25360394 PMC4211261

[B23] Devraj K. Poznanovic S. Spahn C. Schwall G. Harter P. N. Mittelbronn M.. (2016). BACE-1 is expressed in the blood-brain barrier endothelium and is upregulated in a murine model of Alzheimer’s disease. J. Cereb. Blood Flow Metab. 36, 1281–1294. 10.1177/0271678X15606463 26661166 PMC4929696

[B24] Eriksson S. Janciauskiene S. Lannfelt L. (1995). Alpha 1-antichymo-trypsin regulates Alzheimer beta-amyloid peptide fibril formation. Proc. Natl. Acad. Sci. U S A 92, 2313–2317. 10.1073/pnas.92.6.2313 7892264 PMC42805

[B25] Felsky D. Szeszko P. Yu L. Honer W. G. De Jager P. L. Schneider J. A.. (2014). The SORL1 gene and convergent neural risk for Alzheimer’s disease across the human lifespan. Mol. Psychiatry 19, 1125–1132. 10.1038/mp.2013.142 24166411 PMC4004725

[B26] Ferrer I. Martí E. Tortosa A. Blasi J. (1998). Dystrophic neurites of senile plaques are defective in proteins involved in exocytosis and neurotransmission. J. Neuropathol. Exp. Neurol. 57, 218–225. 10.1097/00005072-199803000-00002 9600213

[B27] Finan G. M. Okada H. Kim T. W. (2011). BACE1 retrograde trafficking is uniquely regulated by the cytoplasmic domain of sortilin. J. Biol. Chem. 286, 12602–12616. 10.1074/jbc.M110.170217 21245145 PMC3069461

[B28] Garai K. Verghese P. B. Baban B. Holtzman D. M. Frieden C. (2014). The binding of apolipoprotein E to oligomers and fibrils of amyloid-β alters the kinetics of amyloid aggregation. Biochemistry 53, 6323–6331. 10.1021/bi5008172 25207746 PMC4196732

[B29] García-Marín V. García-López P. Freire M. (2007). Cajal’s contributions to the study of Alzheimer’s disease. J. Alzheimers Dis. 12, 161–174. 10.3233/jad-2007-12206 17917161

[B30] Gelling C. L. Dawes I. W. Perlmutter D. H. Fisher E. A. Brodsky J. L. (2012). The endosomal protein-sorting receptor sortilin has a role in trafficking α-1 antitrypsin. Genetics 192, 889–903. 10.1534/genetics.112.143487 22923381 PMC3522165

[B31] Glenner G. G. Wong C. W. (1984). Alzheimer’s disease: initial report of the purification and characterization of a novel cerebrovascular amyloid protein. Biochem. Biophys. Res. Commun. 120, 885–890. 10.1016/s0006-291x(84)80190-4 6375662

[B32] Gonatas N. K. Anderson W. Evangelista I. (1967). The contribution of altered synapses in the senile plaque: an electron microscopic study in Alzheimer’s dementia. J. Neuropathol. Exp. Neurol. 26, 25–39. 10.1097/00005072-196701000-00003 6022163

[B33] Griffith C. M. Xie M. X. Qiu W. Y. Sharp A. A. Ma C. Pan A. (2016). Aberrant expression of the pore-forming K_ATP_ channel subunit Kir6.2 in hippocampal reactive astrocytes in the 3×Tg-AD mouse model and human Alzheimer’s disease. Neuroscience 336, 81–101. 10.1016/j.neuroscience.2016.08.034 27586053

[B34] Hemming M. L. Elias J. E. Gygi S. P. Selkoe D. J. (2009). Identification of beta-secretase (BACE1) substrates using quantitative proteomics. PLoS One 4:e8477. 10.1371/journal.pone.0008477 20041192 PMC2793532

[B35] Herholz K. Ebmeier K. (2011). Clinical amyloid imaging in Alzheimer’s disease. Lancet Neurol. 10, 667–670. 10.1016/S1474-4422(11)70123-5 21683932

[B36] Hermey G. (2009). The Vps10p-domain receptor family. Cell. Mol. Life Sci. 66, 2677–2689. 10.1007/s00018-009-0043-1 19434368 PMC11115710

[B37] Herms J. Dorostkar M. M. (2016). Dendritic spine pathology in neurodegenerative diseases. Annu. Rev. Pathol. 11, 221–250. 10.1146/annurev-pathol-012615-044216 26907528

[B38] Hsiao K. Chapman P. Nilsen S. Eckman C. Harigaya Y. Younkin S.. (1996). Correlative memory deficits, Aβ elevation and amyloid plaques in transgenic mice. Science 274, 99–102. 10.1126/science.274.5284.99 8810256

[B39] Hu F. Padukkavidana T. Vægter C. B. Brady O. A. Zheng Y. Mackenzie I. R.. (2010). Sortilin-mediated endocytosis determines levels of the frontotemporal dementia protein, progranulin. Neuron 68, 654–667. 10.1016/j.neuron.2010.09.034 21092856 PMC2990962

[B40] Jellinger K. A. Bancher C. (1988). Neuropathology of Alzheimer’s disease: a critical update. J. Neural. Transm. Suppl. 54, 77–95. 10.1007/978-3-7091-7508-8_8 9850917

[B41] Karran E. De Strooper B. (2016). The amyloid cascade hypothesis: are we poised for success or failure? J. Neurochem. 139, 237–252. 10.1111/jnc.13632 27255958

[B42] Kida E. Choi-Miura N. H. Wisniewski K. E. (1995). Deposition of apolipoproteins E and J in senile plaques is topographically determined in both Alzheimer’s disease and down’s syndrome brain. Brain Res. 685, 211–216. 10.1016/0006-8993(95)00482-6 7583250

[B43] Kjolby M. Nielsen M. S. Petersen C. M. (2015). Sortilin, encoded by the cardiovascular risk gene SORT1 and its suggested functions in cardiovascular disease. Curr. Atheroscler. Rep. 17:496. 10.1007/s11883-015-0496-7 25702058

[B44] Louwersheimer E. Ramirez A. Cruchaga C. Becker T. Kornhuber J. Peters O.. (2015). Influence of genetic variants in SORL1 gene on the manifestation of Alzheimer’s disease. Neurobiol. Aging 36, 1605.e13–1605.20. 10.1016/j.neurobiolaging.2014.12.007 25659857

[B45] Luse S. A. Smith K. R. Jr. (1964). The ultrastructure of senile plaques. Am. J. Pathol. 44, 553–563. 5877505 PMC1907027

[B46] Masliah E. Hansen L. Albright T. Mallory M. Terry R. D. (1991). Immunoelectron microscopic study of synaptic pathology in Alzheimer’s disease. Acta Neuropathol. 81, 428–433. 10.1007/bf00293464 1903014

[B47] Masters C. L. Simms G. Weinman N. A. Multhaup G. McDonald B. L. Beyreuther K. (1985). Amyloid plaque core protein in Alzheimer disease and down syndrome. Proc. Natl. Acad. Sci. U S A 82, 4245–4249. 10.1073/pnas.82.12.4245 3159021 PMC397973

[B48] Mathis C. A. Bacskai B. J. Kajdasz S. T. McLellan M. E. Frosch M. P. Hyman B. T.. (2002). A lipophilic thioflavin-T derivative for positron emission tomography (PET) imaging of amyloid in brain. Bioorg. Med. Chem. Lett. 12, 295–298. 10.1016/S0960-894X(01)00734-X 11814781

[B49] Mazella J. Zsürger N. Navarro V. Chabry J. Kaghad M. Caput D.. (1998). The 100-kDa neurotensin receptor is gp95/sortilin, a non-G-protein-coupled receptor. J. Biol. Chem. 273, 26273–26276. 10.1074/jbc.273.41.26273 9756851

[B50] Molgaard S. Demontis D. Nicholson A. M. Finch N. A. Petersen R. C. Petersen C. M.. (2016). Soluble sortilin is present in excess and positively correlates with progranulin in CSF of aging individuals. Exp. Gerontol. 84, 96–100. 10.1016/j.exger.2016.09.002 27612602

[B51] Montine T. J. Phelps C. H. Beach T. G. Bigio E. H. Cairns N. J. Dickson D. W.. (2012). National institute on aging-Alzheimer’s association guidelines for the neuropathologic assessment of Alzheimer’s disease: a practical approach. Acta Neuropathol. 123, 1–11. 10.1007/s00401-011-0910-3 22101365 PMC3268003

[B52] Mortensen M. B. Kjolby M. Gunnersen S. Larsen J. V. Palmfeldt J. Falk E.. (2014). Targeting sortilin in immune cells reduces proinflammatory cytokines and atherosclerosis. J. Clin. Invest. 124, 5317–5322. 10.1172/JCI76002 25401472 PMC4348947

[B53] Mufson E. J. Wuu J. Counts S. E. Nykjaer A. (2010). Preservation of cortical sortilin protein levels in MCI and Alzheimer’s disease. Neurosci. Lett. 471, 129–133. 10.1016/j.neulet.2010.01.023 20085800 PMC2829104

[B54] Navarro V. Vincent J. P. Mazella J. (2002). Shedding of the luminal domain of the neurotensin receptor-3/sortilin in the HT29 cell line. Biochem. Biophys. Res. Commun. 29, 760–764. 10.1016/s0006-291x(02)02564-0 12419319

[B55] Nielsen M. S. Madsen P. Christensen E. I. Nykjaer A. Gliemann J. Kasper D.. (2001). The sortilin cytoplasmic tail conveys golgi-endosome transport and binds the VHS domain of the GGA2 sorting protein. EMBO J. 20, 2180–2190. 10.1093/emboj/20.9.2180 11331584 PMC125444

[B56] Nyborg A. C. Ladd T. B. Zwizinski C. W. Lah J. J. Golde T. E. (2006). Sortilin, SorCS1b and SorLA Vps10p sorting receptors, are novel gamma-secretase substrates. Mol. Neurodegener. 1:3. 10.1186/1750-1326-1-3 16930450 PMC1513133

[B57] Nykjaer A. Lee R. Teng K. K. Jansen P. Madsen P. Nielsen M. S.. (2004). Sortilin is essential for proNGF-induced neuronal cell death. Nature 427, 843–848. 10.1038/nature02319 14985763

[B58] Oakley H. Cole S. L. Logan S. Maus E. Shao P. Craft J.. (2006). Intraneuronal β-amyloid aggregates, neurodegeneration and neuron loss in transgenic mice with 5 familial Alzheimer’s disease mutations: potential factors in amyloid plaque formation. J. Neurosci. 26, 10129–10140. 10.1523/JNEUROSCI.1202-06.2006 17021169 PMC6674618

[B59] Oddo S. Caccamo A. Shepherd J. D. Murphy M. P. Golde T. E. Kayed R.. (2003). Triple-transgenic model of Alzheimer’s disease with plaques and tangles: intracellular Aβ and synaptic dysfunction. Neuron 39, 409–421. 10.1016/S0896-6273(03)00434-3 12895417

[B60] Ogawa K. Ueno T. Iwasaki T. Kujiraoka T. Ishihara M. Kunimoto S.. (2016). Soluble sortilin is released by activated platelets and its circulating levels are associated with cardiovascular risk factors. Atherosclerosis 249, 110–115. 10.1016/j.atherosclerosis.2016.03.041 27085161

[B61] Ohry A. Buda O. (2015). Teofil Simchowicz (1879–1957): the scientist who coined senile plaques in neuropathology. Rom. J. Morphol. Embryol. 56, 1545–1548. Available online at: http://www.rjme.ro/RJME/resources/files/56041515451548.pdf 26743308

[B62] Oĭfa A. I. (1973). Paul Divry—founder of the concept of cerebral amyloidosis. Zh. Nevropatol. Psikhiatr. Im. S S Korsakova 73, 1078–1082. 4591419

[B63] Petersen C. M. Nielsen M. S. Nykjaer A. Jacobsen L. Tommerup N. Rasmussen H. H.. (1997). Molecular identification of a novel candidate sorting receptor purified from human brain by receptor-associated protein affinity chromatography. J. Biol. Chem. 272, 3599–3605. 10.1074/jbc.272.6.3599 9013611

[B64] Poo M. M. Du J. L. Ip N. Y. Xiong Z. Q. Xu B. Tan T. (2016). China brain project: basic neuroscience, brain diseases and brain-inspired computing. Neuron 92, 591–596. 10.1016/j.neuron.2016.10.050 27809999

[B65] Pottier C. Hannequin D. Coutant S. Rovelet-Lecrux A. Wallon D. Rousseau S.. (2012). High frequency of potentially pathogenic SORL1 mutations in autosomal dominant early-onset alzheimer disease. Mol. Psychiatry 17, 875–879. 10.1038/mp.2012.15 22472873

[B66] Reitz C. Tokuhiro S. Clark L. N. Conrad C. Vonsattel J. P. Hazrati L. N.. (2011). SORCS1 alters amyloid precursor protein processing and variants may increase Alzheimer’s disease risk. Ann. Neurol. 69, 47–64. 10.1002/ana.22308 21280075 PMC3086759

[B67] Robakis N. K. Wisniewski H. M. Jenkins E. C. Devine-Gage E. A. Houck G. E. Yao X. L.. (1987). Chromosome 21q21 sublocalization of gene encoding β-amyloid peptide in cerebral vessels and neuritic (senile) plaques of people with Alzheimer disease and down syndrome. Lancet 1, 384–385. 2880184 10.1016/s0140-6736(87)91754-5

[B68] Rogaeva E. Meng Y. Lee J. H. Gu Y. Kawarai T. Zou F.. (2007). The neuronal sortilin-related receptor SORL1 is genetically associated with Alzheimer disease. Nat. Genet. 39, 168–177. 10.1038/ng1943 17220890 PMC2657343

[B69] Rogers J. Cooper N. R. Webster S. Schultz J. McGeer P. L. Styren S. D.. (1992). Complement activation by β-amyloid in Alzheimer disease. Proc. Natl. Acad. Sci. U S A 89, 10016–10020. 1438191 10.1073/pnas.89.21.10016 PMC50268

[B70] Ruan C. S. Yang C. R. Li J. Y. Luo H. Y. Bobrovskaya L. Zhou X. F. (2016). Mice with sort1 deficiency display normal cognition but elevated anxiety-like behavior. Exp. Neurol. 281, 99–108. 10.1016/j.expneurol.2016.04.015 27118371

[B71] Saadipour K. Yang M. Lim Y. Georgiou K. Sun Y. Keating D.. (2013). Amyloid beta (Aβ) up-regulates the expression of sortilin via the p75 (NTR)/RhoA signaling pathway. J. Neurochem. 127, 152–162. 10.1111/jnc.12383 23895422

[B72] Sadleir K. R. Kandalepas P. C. Buggia-Prévot V. Nicholson D. A. Thinakaran G. Vassar R. (2016). Presynaptic dystrophic neurites surrounding amyloid plaques are sites of microtubule disruption, BACE1 elevation and increased Aβ generation in Alzheimer’s disease. Acta Neuropathol. 132, 235–256. 10.1007/s00401-016-1558-9 26993139 PMC4947125

[B73] Sarret P. Krzywkowski P. Segal L. Nielsen M. S. Petersen C. M. Mazella J.. (2003). Distribution of NTS3 receptor/sortilin mRNA and protein in the rat central nervous system. J. Comp. Neurol. 461, 483–505. 10.1002/cne.10708 12746864

[B74] Schwarzman A. L. Gregori L. Vitek M. P. Lyubski S. Strittmatter W. J. Enghilde J. J.. (1994). Transthyretin sequesters amyloid beta protein and prevents amyloid formation. Proc. Natl. Acad. Sci. U S A 91, 8368–8372. 10.1016/0197-4580(94)92672-7 8078889 PMC44607

[B75] Shea Y. F. Chu L. W. Chan A. O. Ha J. Li Y. Song Y. Q. (2016). A systematic review of familial Alzheimer’s disease: differences in presentation of clinical features among 3 mutated genes and potential ethnic differences. J. Formos. Med. Assoc. 115, 67–75. 10.1016/j.jfma.2015.08.004 26337232

[B76] Strong A. Ding Q. Edmondson A. C. Millar J. S. Sachs K. V. Li X.. (2012). Hepatic sortilin regulates both apolipoprotein B secretion and LDL catabolism. J. Clin. Invest. 122, 2807–2816. 10.1172/JCI63563 22751103 PMC3408750

[B77] Struble R. G. Cork L. C. Whitehouse P. J. Price D. L. (1982). Cholinergic innervation in neuritic plaques. Science 216, 413–415. 10.1126/science.6803359 6803359

[B78] Struble R. G. Powers R. E. Casanova M. F. Kitt C. A. Brown E. C. Price D. L. (1987). Neuropeptidergic systems in plaques of Alzheimer’s disease. J. Neuropathol. Exp. Neurol. 46, 567–584. 2442313 10.1097/00005072-198709000-00006

[B79] Vassar R. Kovacs D. M. Yan R. Wong P. C. (2009). The β-secretase enzyme BACE in health and Alzheimer’s disease: regulation, cell biology, function and therapeutic potential. J. Neurosci. 29, 12787–12794. 10.1523/JNEUROSCI.3657-09.2009 19828790 PMC2879048

[B80] Verheijen J. Van den Bossche T. van der Zee J. Engelborghs S. Sanchez-Valle R. Lladó A.. (2016). A comprehensive study of the genetic impact of rare variants in SORL1 in European early-onset Alzheimer’s disease. Acta Neuropathol. 132, 213–224. 10.1007/s00401-016-1566-9 27026413 PMC4947104

[B81] Walker L. C. Kitt C. A. Struble R. G. Schmechel D. E. Oertel W. H. Cork L. C.. (1985). Glutamic acid decarboxylase-like immunoreactive neurites in senile plaques. Neurosci. Lett. 59, 165–169. 10.1016/0304-3940(85)90194-6 2997667

[B82] Watson M. D. Roher A. E. Kim K. S. Spiegel K. Emmerling M. (1997). Complement interactions with amyloid β1–42: a nidus for inflammation in AD brains. Amyloid 4, 147–156. 10.3109/13506129709014379

[B83] Wen Y. Miyashita A. Kitamura N. Tsukie T. Saito Y. Hatsuta H.. (2013). SORL1 is genetically associated with neuropathologically characterized late-onset Alzheimer’s disease. J. Alzheimers Dis. 35, 387–394. 10.3233/JAD-122395 23455993

[B84] Westergaard U. B. Sørensen E. S. Hermey G. Nielsen M. S. Nykjaer A. Kirkegaard K.. (2004). Functional organization of the sortilin Vps10p domain. J. Biol. Chem. 279, 50221–50229. 10.1074/jbc.M408873200 15364913

[B85] Wolfe M. S. Haass C. (2001). The role of presenilins in gamma-secretase activity. J. Biol. Chem. 276, 5413–5416. 10.1074/jbc.R000026200 11134059

[B86] Wu C. W. Liao P. C. Yu L. Wang S. T. Chen S. T. Wu M.. (2004). Hemoglobin promotes Aβ oligomer formation and localizes in neurons and amyloid deposits. Neurobiol. Dis. 17, 367–377. 10.1016/j.nbd.2004.08.014 15571973

[B87] Xue Z. Q. He Z. W. Yu J. J. Cai Y. Qiu W. Y. Pan A.. (2015). Non-neuronal and neuronal BACE1 elevation in association with angiopathic and leptomeningeal β-amyloid deposition in the human brain. BMC Neurol. 15:71. 10.1186/s12883-015-0327-z 25934480 PMC4428107

[B88] Yamaguchi H. Hirai S. Morimatsu M. Shoji M. Ihara Y. (1988). A variety of cerebral amyloid deposits in the brains of the Alzheimer-type dementia demonstrated by beta protein immunostaining. Acta Neuropathol. 76, 541–549. 10.1007/bf00689591 3059748

[B89] Yan X. X. Ma C. Gai W. P. Cai H. Luo X. G. (2014). Can BACE1 inhibition mitigate early axonal pathology in neurological diseases? J. Alzheimers Dis. 38, 705–718. 10.3233/JAD-131400 24081378 PMC3995167

[B90] Yan X. X. Ma C. Bao A. M. Wang X. M. Gai W. P. (2015). Brain banking as a cornerstone of neuroscience in China. Lancet Neurol. 14:136. 10.1016/s1474-4422(14)70259-5 25772887

[B91] Yang M. Lim Y. Li X. Zhong J. H. Zhou X. F. (2011). Precursor of brain-derived neurotrophic factor (proBDNF) forms a complex with huntingtin-associated protein-1 (HAP1) and sortilin that modulates proBDNF trafficking, degradation and processing. J. Biol. Chem. 286, 16272–16284. 10.1074/jbc.M110.195347 21357693 PMC3091234

[B92] Yang M. Virassamy B. Vijayaraj S. L. Lim Y. Saadipour K. Wang Y. J.. (2013). The intracellular domain of sortilin interacts with amyloid precursor protein and regulates its lysosomal and lipid raft trafficking. PLoS One 8:e63049. 10.1371/journal.pone.0063049 23704887 PMC3660575

[B93] Zeng F. Deng Y. P. Yi X. Cao H. Y. Zou H. Q. Wang X.. (2013). No association of SORT1 gene polymorphism with sporadic Alzheimer’s disease in the chinese han population. Neuroreport 24, 464–468. 10.1097/WNR.0b013e3283619f43 23660633

[B94] Zhang X. M. Cai Y. Xiong K. Cai H. Luo X. G. Feng J. C.. (2009). Beta-secretase-1 elevation in transgenic mouse models of Alzheimer’s disease is associated with synaptic/axonal pathology and amyloidogenesis: implications for neuritic plaque development. Eur. J. Neurosci. 30, 2271–2283. 10.1111/j.1460-9568.2009.07017.x 20092570 PMC2869535

[B95] Zhu H. X. Xue Z. Q. Qiu W. Y. Zeng Z. J. Dai J. P. Ma C.. (2015). Age-related intraneuronal accumulation of αII-spectrin breakdown product SBDP120 in the human cerebrum is enhanced in Alzheimer’s disease. Exp. Gerontol. 69, 43–52. 10.1016/j.exger.2015.06.003 26051930

