# Supplementary material for: Sortilin Fragments Deposit at Senile Plaques in Human Cerebrum
Source: Front Neuroanat. 2017 Jun 7;11:45. doi: 10.3389/fnana.2017.00045 (PMC5461299; doi:10.3389/fnana.2017.00045)
Supplement: Supplementary file 1 [file Table_1.pdf]

Supplementary Table 1: Postmortem human brain samples used for comparative anatomic and biochemical analyses in this study#

Grouping		Case #	Sex	Age (yrs)	Postmortem delay (hrs)	Clinical diagnosis or cause of death	Tauopathy	Amyloid pathology	Tissue utility
Cases used for pathological and immunoblot characterization	AD/demented group (age: 87 ±9.8 years)*	Case #1	M	92	5.5	Respiratory failure, demented**	V	C	IHC/WB
		Case #2	M	85	4.5	Pneumonia, demented	VI	C	IHC/WB/IF***
		Case #3	F	80	22	AD	VI	C	IHC/WB
		Case #4	M	95	5	Internal bleeding, demented	VI	C	IHC/WB
		Case #5	F	100	3	Hypertension, demented	VI	C	IHC/WB/IF
		Case #6	F	80	5.5	AD	IV	C	IHC/WB/IF
		Case #7	F	81	5.5	AD	IV	C	IHC/WB
		Case #8	F	99	6.3	Cerebral stroke, demented	III	B	IHC/WB/IF
		Case #9	M	72	4.5	Multisystem failure, demented	V	C	IHC/WB/IF
	Aged group (age = 80 ±4.6 years)*	Case #1	M	73	6.5	Cardiac stroke	(-)****	(-)	IHC/WB
		Case #2	M	80	4.5	Multisystem failure, bone cancer	I	(-)	IHC/WB
		Case #3	F	85	7.5	Liver cancer	(-)	(-)	IHC/WB
		Case #4	F	86	6.5	Multisystem failure, demented	II	(-)	IHC/WB
		Case #5	F	80	18	Sjogren's syndrome, demented	(-)	(-)	IHC/WB
		Case #6	M	75	5.3	Prostate cancer	(-)	(-)	IHC/WB
		Case #7	M	85	5	Cardiac stroke	II	(-)	IHC/WB
		Case #8	M	77	20	Colon cancer	(-)	(-)	IHC/WB
		Case #9	M	79	6	Pneumonia	(-)	(-)	IHC/WB
	Mid-age group (age = 56 ±8.2 years)	Case #1	F	59	5	Respiratory failure	(-)	(-)	IHC/WB
		Case #2	M	61	8.5	Lung cancer	(-)	(-)	IHC/WB
		Case #3	F	47	4.5	Cerebral stroke	(-)	(-)	IHC/WB
		Case #4	F	43	13.5	Liver cancer	(-)	(-)	IHC/WB
		Case #5	F	48	5.5	Leukemia	(-)	(-)	IHC/WB
		Case #6	M	59	12	Colon cancer	(-)	(-)	IHC/WB
		Case #7	M	65	30.5	Cardiac failure	(-)	(-)	IHC/WB
		Case #8	F	62	16	Lung cancer	(-)	(-)	IHC/WB
		Case #9	F	64	4.5	Ovarian cancer	(-)	(-)	IHC/WB

Cases used for comparative pathological characterization	Case #1	M	79	13	Lung cancer	V	C	IHC/IF
	Case #2	M	98	7	Pneumonia	III	B	IHC/IF
	Case #3	F	80	4.5	Cerebral stroke, demented	VI	C	IHC/IF
	Case #4	M	89	5	Pneumonia, demented	VI	C	IHC/IF
	Case #5	F	71	8.5	Breast cancer	III	B	IHC/IF
	Case #6	F	78	19	Cardiac stroke	IV	C	IHC/IF
	Case #7	M	95	4	Pneumonia	V	C	IHC
	Case #8	M	89	4.5	Gall bladder cancer	IV	B	IHC
	Case #9	F	87	5	Pneumonia	V	B	IHC
	Case #10	F	96	6	Pneumonia, urinary tract cancer	II	B	IHC
	Case #11	M	94	5	Diabetes mellitus	III	B	IHC
	Case #12	M	86	6	Hypertension	II	B	IHC
	Case #13	M	89	3	Multisystem failure	II	(-)	IHC
	Case #14	F	87	6	Hypertension	II	(-)	IHC
	Case #15	F	95	5.5	Pneumonia	I	B	IHC
	Case #16	M	78	16.5	Prostate cancer	II	A	IHC
	Case #17	M	70	5.3	Cardiac stroke	II	B	IHC/IF
	Case #18	M	87	8	Stomach Cancer	II	B	IHC
	Case #19	M	58	4	Multisystem failure	(-)	(-)	IHC
	Case #20	M	67	7.5	Lung cancer	(-)	(-)	IHC
	Case #21	M	79	5	Cerebral stroke	(-)	(-)	IHC
	Case #22	F	76	8	Lung cancer	(-)	(-)	IHC
	Case #23	M	59	40	Stomach Cancer	(-)	(-)	IHC
	Case #24	M	35	7	Hepatic Cancer	(-)	(-)	IHC
	Case #25	F	68	4.5	Glioma	(-)	(-)	IHC
	Case #26	F	70	18	Septicemia	(-)	(-)	IHC
	Case #27	M	72	26.5	Septicemia	(-)	(-)	IHC
	Case #28	M	71	8	Cerebral stroke	(-)	(-)	IHC

#Pathologically examined samples from clinically diagnosed AD cases from Rush University Hospital are not listed

*P value=0.066 by paired Student-t test

**Demented: information provided by caregivers at brain donation

***IHC: Immunohistochemistry; WB: western blot; IF: immunofluorescence

****(-): Amyloid or Tau pathology not observed in microscopic examination of immunohistochemical preparations
