# Supplementary material for: Sortilin Fragments Deposit at Senile Plaques in Human Cerebrum
Source: Front Neuroanat. 2017 Jun 7;11:45. doi: 10.3389/fnana.2017.00045 (PMC5461299; doi:10.3389/fnana.2017.00045)
Supplement: Supplementary file 2 [file Image_1.pdf]

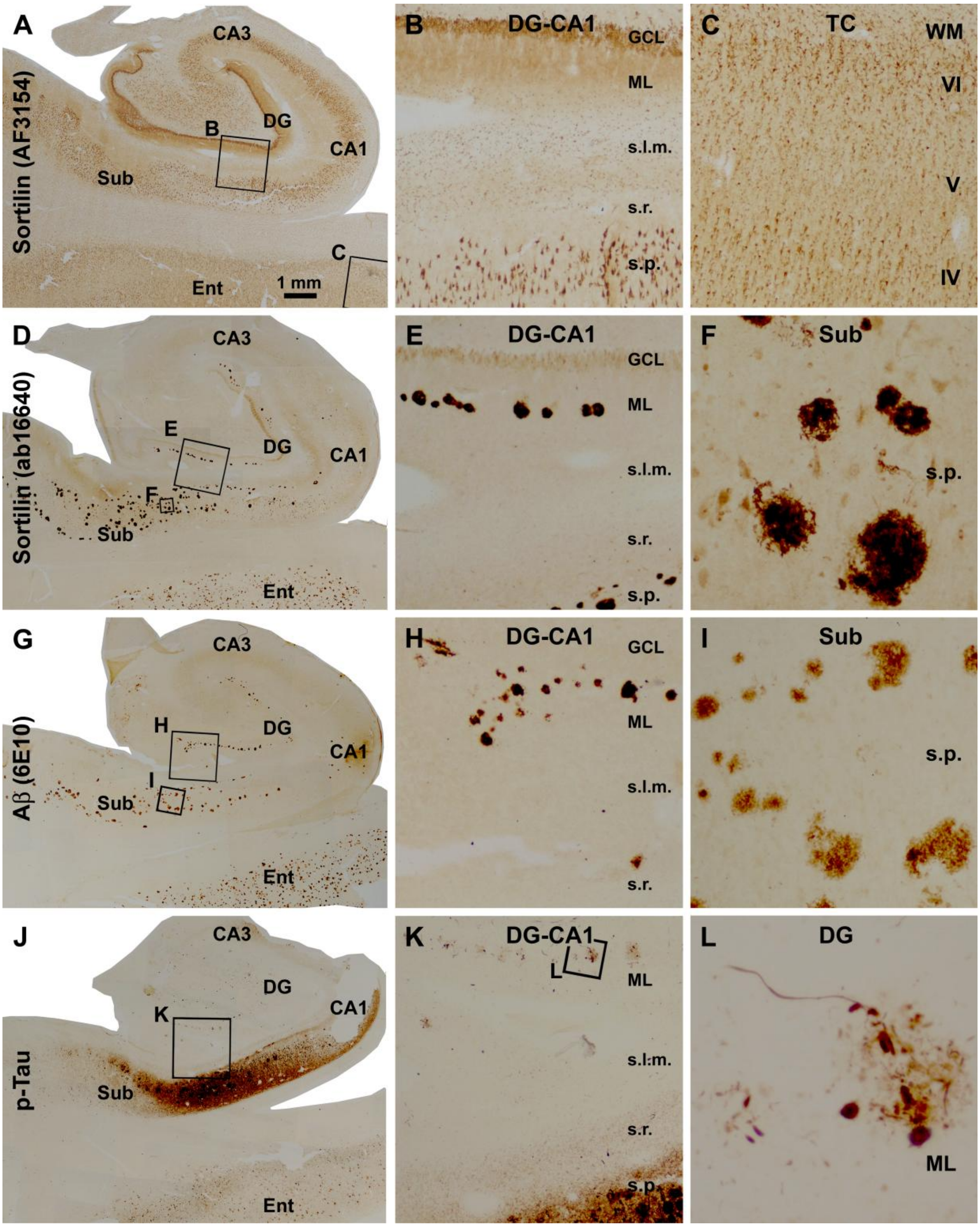

Supplemental Figure 1. Morphological characterization of sortilin labeled plaque lesions relative to amyloid and tau pathology using sections from an additional case with Alzheimer's disease (AD). Panels (A-C) show low and enlarged (framed areas) views of the exclusive neuronal labeling with the goat sortilin antibody over the hippocampal formation and part of temporal cortex (TC). Panels (D-F) show the rabbit sortilin antibody labeling of plaque lesions as well as neurons in an adjacent section. Panels (G-I) illustrate 6E10 labeling of β-amyloid (Aβ) deposition in the same temporal lobe regions, with the laminar/regional distribution (G) of the plaques matched to that labeled by the rabbit sortilin antibody seen in (D). Panels (J-L) show phosphorylated tau (p-Tau) labeling across the regions, extremely intense in the subiculum. Enlarged panels from the framed areas show the morphological details of labeled profiles. Abbreviations are as defined in Fig. 1. Scale bar = 1 mm in (A) applying to (D, G, J); equivalent to 200 μm for (B, C, E, H, K), 50 μm for (F, I) and 25 μm for (L).
