# Supplementary material for: Sortilin Fragments Deposit at Senile Plaques in Human Cerebrum
Source: Front Neuroanat. 2017 Jun 7;11:45. doi: 10.3389/fnana.2017.00045 (PMC5461299; doi:10.3389/fnana.2017.00045)
Supplement: Supplementary file 3 [file Image_2.pdf]

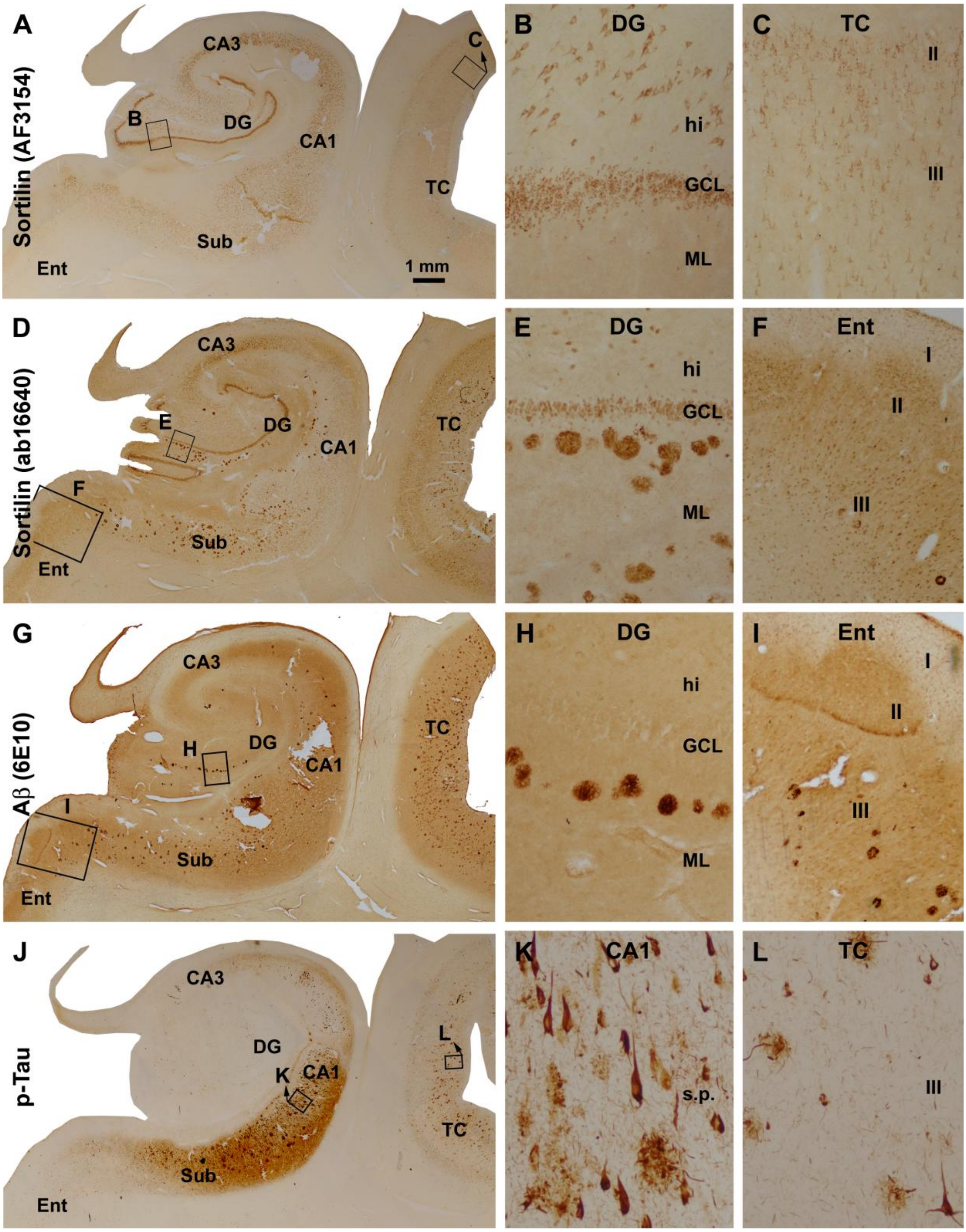


**Supplemental Figure 2.** Morphological characterization of sortilin labeled plaques relative to Aβ and tau pathology using sections from an aged case not demented at the time hospitalized for the care of terminal illness. Panels (A-C) show low and enlarged (framed areas) views of labeling with the goat sortilin antibody, in the hippocampal and neocortical neurons of the temporal lobe. Panels (D-F) show labeling with the rabbit antibody at plaque lesions and neuronal profiles in an adjacent section. Panels (G-I) illustrate deposition labeled by 6E10 over the same temporal lobe areas. Dense-packing sortilin and Aβ plaques exhibit similar distribution pattern across the temporal lobe structures (E, E, G, H), while diffuse Aβ deposition at the layer II cell islands in the entorhinal cortex is not associated with extracellular sortilin labeling (F, I). Panels (J-L) show p-Tau immunolabeling at neuritic plaques and in tangled neurons and neuronal processes at high magnification. Abbreviations are as defined in Fig. 1. Scale bar = 1 mm in (A) applying to (D, G, J); equivalent to 500 µm for (F, I), 200 µm for (B, C, E, H) and 50 µm for (K, L).
